# Drivers of Natural Variation in Water-Use Efficiency Under Fluctuating Light Are Promising Targets for Improvement in Sorghum

**DOI:** 10.3389/fpls.2021.627432

**Published:** 2021-02-01

**Authors:** Charles P. Pignon, Andrew D. B. Leakey, Stephen P. Long, Johannes Kromdijk

**Affiliations:** ^1^Department of Plant Biology, University of Illinois at Urbana-Champaign, Urbana, IL, United States; ^2^Department of Crop Sciences, University of Illinois at Urbana-Champaign, Urbana, IL, United States; ^3^Carl R. Woese Institute for Genomic Biology, University of Illinois at Urbana-Champaign, Urbana, IL, United States; ^4^Lancaster Environment Centre, Lancaster University, Lancaster, United Kingdom; ^5^Department of Plant Sciences, University of Cambridge, Cambridge, United Kingdom

**Keywords:** water-use efficiency, stomata, photosynthesis, non-steady-state gas-exchange, fluctuating light, dynamic light, sustainability, sorghum

## Abstract

Improving leaf intrinsic water-use efficiency (*iWUE*), the ratio of photosynthetic CO_2_ assimilation to stomatal conductance, could decrease crop freshwater consumption. *iWUE* has primarily been studied under steady-state light, but light in crop stands rapidly fluctuates. Leaf responses to these fluctuations substantially affect overall plant performance. Notably, photosynthesis responds faster than stomata to decreases in light intensity: this desynchronization results in substantial loss of *iWUE*. Traits that could improve *iWUE* under fluctuating light, such as faster stomatal movement to better synchronize stomata with photosynthesis, show significant natural diversity in C_3_ species. However, C_4_ crops have been less closely investigated. Additionally, while modification of photosynthetic or stomatal traits independent of one another will theoretically have a proportionate effect on *iWUE*, in reality these traits are inter-dependent. It is unclear how interactions between photosynthesis and stomata affect natural diversity in *iWUE*, and whether some traits are more tractable drivers to improve *iWUE*. Here, measurements of photosynthesis, stomatal conductance and *iWUE* under steady-state and fluctuating light, along with stomatal patterning, were obtained in 18 field-grown accessions of the C_4_ crop sorghum. These traits showed significant natural diversity but were highly correlated, with important implications for improvement of *iWUE*. Some features, such as gradual responses of photosynthesis to decreases in light, appeared promising for improvement of *iWUE*. Other traits showed tradeoffs that negated benefits to *iWUE*, e.g., accessions with faster stomatal responses to decreases in light, expected to benefit *iWUE*, also displayed more abrupt losses in photosynthesis, resulting in overall lower *iWUE*. Genetic engineering might be needed to break these natural tradeoffs and achieve optimal trait combinations, e.g., leaves with fewer, smaller stomata, more sensitive to changes in photosynthesis. Traits describing *iWUE* at steady-state, and the change in *iWUE* following decreases in light, were important contributors to overall *iWUE* under fluctuating light.

## Introduction

Water is the primary abiotic factor limiting crop productivity (Boyer, 1982), with agriculture consuming up to 85% of freshwater withdrawals (WWAP, 2015; D’Odorico et al., 2020). Breeding has almost tripled productivity of major crops over the last 60 years, without parallel improvement in the amount of water required to produce a ton of crop biomass (Sinclair et al., 1984; Ort and Long, 2014). With changing patterns of precipitation and increased drought frequency (Dai, 2013; Spinoni et al., 2018; Sun et al., 2018), rising atmospheric vapor pressure deficit due to global warming (Ort and Long, 2014), and decreasing groundwater supply around the world (Dalin et al., 2017), supplying sufficient water to crops is increasingly difficult and unsustainable (Lobell et al., 2014; WWAP, 2015). Therefore, improving crop water-use efficiency is important to achieve the crop productivity required to meet global demand (Bonsch et al., 2016; Flexas, 2016; FAO et al., 2018; Leakey et al., 2019).

The dependence of crop productivity on water supply derives from the interaction between leaf photosynthetic CO_2_ assimilation (*A*) and transpiration. Stomatal pores on the leaf surface allow CO_2_ diffusion into the leaf for *A*, but also allow water vapor to escape from the leaf. The inverse of the resistance to water vapor loss collectively imposed by the stomatal pores is measured as the stomatal conductance to water vapor (*g*_*s*_). The ratio of *A* to *g*_*s*_ gives leaf intrinsic water-use efficiency (*iWUE*) (Leakey et al., 2019). Most inter- and intra-specific surveys of *A*, *g*_*s*_ and *iWUE*, and analyses of their limitations have concerned steady-state conditions (Galmes et al., 2007; Giuliani et al., 2013; Driever et al., 2014; Jahan et al., 2014; Sollenberger et al., 2014; Viswanathan et al., 2014; Tomeo and Rosenthal, 2017; Yabiku and Ueno, 2017; Leakey et al., 2019).

Although steady-state measurements are important to understand plant physiological performance (von Caemmerer and Farquhar, 1981; von Caemmerer, 2000), leaves in field-grown crop canopies are rarely in steady-state light conditions, and experience frequent fluctuations in incident photosynthetic photon flux density (*PPFD*) that cause substantial deviations from steady-state carbon and water fluxes (Pearcy, 1990; Zhu et al., 2004; Kaiser et al., 2015, 2018; McAusland et al., 2016; Vialet-Chabrand et al., 2017; Slattery et al., 2018; Acevedo-Siaca et al., 2020; De Souza et al., 2020; Wang et al., 2020). Evidence of increased water deficit stress (Sun et al., 2017), temperature stress (Leakey et al., 2003), and sensitivity of *A* to elevated CO_2_ (Leakey et al., 2002; Tomimatsu and Tang, 2016) under fluctuating *PPFD*, and recent breakthroughs in improving *A* under fluctuating *PPFD* by manipulating photoprotection (Kromdijk et al., 2016; Hubbart et al., 2018), chlorophyll content (Gu et al., 2017), and stomatal light-sensing (Papanatsiou et al., 2019), have demonstrated the value of considering leaf behavior under non-steady-state lighting conditions.

*A* and *g*_*s*_ typically require several minutes to rise to a new steady-state following an increase in *PPFD*, as in a sun fleck within a crop canopy. The rate of increase in *g*_*s*_ may be mechanically limited by stomatal opening kinetics and stomatal patterning. The rate of increase in *A* may be limited by the kinetics of biochemical processes, in particular Rubisco activation (Soleh et al., 2016, 2017; Acevedo-Siaca et al., 2020), or the kinetics of stomatal opening and the associated mitigation of intercellular CO_2_ (*c*_*i*_)-limitation to *A* (Lawson and Blatt, 2014; McAusland et al., 2016; De Souza et al., 2020). Because the response times of *A* and *g*_*s*_ are similar following an increase in *PPFD*, the deviation of *iWUE* from steady-state is relatively modest (McAusland et al., 2016). In contrast, the response times of *A* and *g*_*s*_ are more distinct following a decrease in *PPFD*, as *A* typically declines to a new steady-state within seconds while *g*_*s*_ decreases over the course of several minutes as stomata close (Lawson et al., 2011; Way and Pearcy, 2012; Lawson and Blatt, 2014; McAusland et al., 2016; Lawson and Vialet-Chabrand, 2019). This lag in *g*_*s*_ relative to *A* leads to continued water loss, resulting in reduced *iWUE*. While the decrease in *A* is often simplified as a step-change from one steady-state to the next (McAusland et al., 2016; Bellasio et al., 2017), physiological processes such as post-illumination decarboxylation of photorespiratory metabolites and slow relaxation of photoprotective mechanisms may temporarily suppress *A* following a decrease in *PPFD* (Doncaster et al., 1989; Kaiser et al., 2015; Kromdijk et al., 2016; Wang et al., 2020).

Attempts to improve leaf physiology under fluctuating light have generally followed three axes: (1) accelerate induction of *A* following increases in *PPFD* (Soleh et al., 2016, 2017; Taylor and Long, 2017; Acevedo-Siaca et al., 2020; Wang et al., 2020), (2) limit the inhibition of *A* following decreases in *PPFD* (Kromdijk et al., 2016; Wang et al., 2020), (3) accelerate stomatal movement to reduce *g*_*s*_ response time, leading to improved *A* following increases in *PPFD* and improved *iWUE* following decreases in *PPFD* (Wang et al., 2014; Papanatsiou et al., 2019; Lawson and Matthews, 2020). An essential component of this research has been the discovery of broad diversity in *A*, *g*_*s*_, and *iWUE* in fluctuating light across species (McAusland et al., 2016; Deans et al., 2019) and within C_3_ species such as rice (Acevedo-Siaca et al., 2020), cassava (De Souza et al., 2020), and soybean (Soleh et al., 2016, 2017; Wang et al., 2020). In contrast, within-species diversity in C_4_ species is not well understood, despite use of the C_4_ pathway by some of the world’s most productive crops, including maize (*Zea mays* L.), sorghum (*Sorghum bicolor* (Lu.) Moench), and sugarcane (*Saccharum officinarum* L.). Further, while modification of *A* or *g*_*s*_ traits independent of one another will theoretically have a proportionate effect on *iWUE*, in reality these traits are inter-dependent. It is unclear how interactions between these traits affect natural diversity in *iWUE*, and whether some traits might be more tractable drivers for improvement of *iWUE*. In C_4_ crops, there is the added complexity of the carbon-concentrating mechanism, which may alter the relationship between *A* and *g*_*s*_ relative to C_3_ species (McAusland et al., 2016). Similarly, it is unclear whether steady-state and non-steady-state traits interact with one another and are predictive of overall leaf gas-exchange under fluctuating light.

Here, steady-state and non-steady-state *iWUE* were examined in the model C_4_ crop sorghum. Sorghum is a key food crop for water-limited regions of the globe and fiber sorghum is among the most productive potential cellulosic bioenergy crops of the warm temperate zone, characterized by high drought-tolerance water-use efficiency and productivity (Regassa and Wortmann, 2014; Hadebe et al., 2017). This study asked three questions:

(1) Is there significant diversity in non-steady-state responses of *A*, *g*_*s*_ and *iWUE* to light fluctuations among diverse sorghum accessions?
(2) What are the main drivers for natural variation in *iWUE* in terms of steady-state *A* and *g*_*s*_, non-steady-state *A* and *g*_*s*_, and stomatal patterning?
(3) Are steady-state and non-steady-state gas-exchange traits predictive of leaf performance under fluctuating *PPFD*?

We analyzed gas exchange and stomatal patterning in 18 diverse sorghum accessions under steady-state and fluctuating *PPFD* conditions. Our results identify drivers for improvement of *A* and *iWUE*, including stomatal size and non-steady-state *A* and *iWUE* traits, and demonstrate that the sorghum pan-genome harbors significant potential for improving the already impressive *iWUE* of sorghum via breeding.

## Materials and Methods

### Plant Material and Growing Conditions

Seeds of 18 sorghum accessions were planted on May 28, 2017 at the University of Illinois Energy Farm near Urbana, IL, United States (40°07′N, 5 88°21′W, 228 m above sea level) in 3 m rows at 25 seeds m^–1^, in plots of four rows spaced 76 cm apart. The accessions were a subset of a biomass sorghum diversity panel that was previously screened to identify transient stomatal responses to a decrease in *PPFD* (Pignon, 2017). Soils are deep silt loam Flanagan and silty clay loam Drummer. Plants were fertilized (101 kg/ha N) and rainfed. In August, the youngest fully expanded leaf was sampled from 3 to 5 plants per accession. Leaves were cut pre-dawn, recut with the cut-end underwater, and transferred to the laboratory for analysis.

### Gas Exchange Measurements

Leaf cuvettes of two portable photosynthetic gas-exchange systems (LI-6400XT; LI-COR, Inc., Lincoln, NE, United States with LI-6400-40 Leaf-Chamber-Fluorometer) were clamped onto each side of the midrib at the middle of each leaf. *PPFD* was set to 2000 μmol m^–2^ s^–1^, block temperature to 25°C, flow rate to 700 μmol s^–1^, [CO_2_] in the sample cell set to 400 ppm and leaf-to-air VPD maintained <2 kPa. LEDs provided 10% blue and 90% red light. Leaves were acclimated to these conditions for an hour, then measurements began.

Because increases in *PPFD* are typically less impactful to *iWUE* than decreases in *PPFD*, this study focused on extracting non-steady-state traits following decreases in *PPFD*. For each leaf, one cuvette measured steady-state *PPFD* response curves, which were used to obtain *A*, *g*_*s*_, and *iWUE* at steady-state, and to evaluate the transition from one steady-state to the next after decreases in *PPFD*. Curves were obtained by decreasing *PPFD* (2000, 1500, 1000, 500, 200, 100, and 0 μmol m^–2^ s^–1^) in 15 min steps, with data recorded every 10 s. Though useful for the purposes of quantifying steady-state and non-steady-state gas-exchange, this *PPFD* timecourse was highly artificial and provided limited information about repeated *PPFD* fluctuations, since it was only composed of lengthy step declines in *PPFD*. Therefore, in the second cuvette, additional measurements were used to determine whether the traits derived from steady-state *PPFD* response curves were predictive of performance in a repeatedly fluctuating *PPFD* environment. Fluctuating *PPFD* response curves were obtained by varying *PPFD* in 5.5 min steps in the following sequence: 2000, 1500, 2000, 1000, 2000, 500, 2000, 200, 2000, and 100 μmol m^–2^ s^–1^, with data recorded every 10 s.

Variation in environmental variables throughout gas-exchange measurements is given in Supplementary Table 1. On completion of gas-exchange the measured leaf tissue was sampled and frozen for stomatal phenotyping.

### Stomatal Phenotyping

Leaf tissue was mounted on glass slides and the abaxial surface was imaged using an optical topometer (μsurf Explorer, NanoFocus, Karlsruhe, Germany) with 20× (0.8 mm^2^ leaf surface, 20× M Plan APO, Olympus Corporation, Tokyo, Japan) and 50× air objectives (0.32 mm^2^ leaf surface, 50× UM Plan FL N, Olympus Corporation). Reconstructions of the leaf epidermis were obtained from serial optical sections measured from surface to inside of leaf, with measurement depth of 40 μm and 20 μm for the 20× and 50× objectives, respectively (μsurf Metrology, NanoFocus). Four images at 20×, and one image at 50×, were taken per leaf. Stomata on 20× images were counted to estimate stomatal density. The size, i.e., planar surface area, of the stomatal complex was measured by outlining relevant pixels of four stomata per 50× image using ImageJ (ImageJ1.51j8, NIH, United States).

### Gas-Exchange Data Analysis

#### Analysis of Steady-State PPFD Response Curves

Steady-state net rate of leaf photosynthetic CO_2_ uptake (*A*_2000_), stomatal conductance to water vapor (*g_*s* 2000_*) and intrinsic water-use efficiency (*iWUE*_2000_) at *PPFD* = 2000 μmol m^–2^ s^–1^ were obtained as the average for the two cuvettes (i.e., two technical reps) for each leaf over the last 40 s of the initial 1 h acclimation period. Steady-state *A*, *g*_*s*_, and *iWUE* were also obtained for each *PPFD* in the steady-state *PPFD* response curve, as the average over the last 40 s of each *PPFD*.

After each decrease in *PPFD*, *A* declined from one steady-state to the next, often displaying a temporary inhibition as it decreased below steady-state (i.e., undershoot), then increased again to steady-state. This process was described by two traits: *t_95 A_* and *A*_*undershoot*_. *t_95 A_* was the time required for *A* to come within 5% of steady-state after a decrease in *PPFD*, where smaller *t_95 A_* indicates that *A* reached steady-state more rapidly. *A*_*undershoot*_ was the difference between steady-state *A* and the minimum *A* reached at a given *PPFD*, where more negative *A*_*undershoot*_ indicates more pronounced undershoot of steady-state. Corresponding traits were used to describe *g*_*s*_ (*t_95 *gs*_*, *g*_*s undershoot*_).

The response of *iWUE* to each decrease in *PPFD* was different from that of *A* and *g*_*s*_, as *iWUE* first abruptly declined, reflecting an instantaneous loss of *A* while *g*_*s*_ remained relatively high. In the following minutes, as *g*_*s*_ declined, *iWUE* increased toward steady-state. *t_95 iWUE_* was calculated analogous to *t_95 A_* and *t_95 gs_*, and *iWUE_*undershoot*_* was calculated analogous to *A*_*undershoot*_ and *g*_*s undershoot*_.

#### Analysis of Fluctuating PPFD Response Curves

Average *A*, *g*_*s*_ and *iWUE* were computed throughout the fluctuating *PPFD* response curves. If responses of *A*, *g*_*s*_, and *iWUE* to changes in *PPFD* were instantaneous, then gas-exchange under fluctuating *PPFD* would be equivalent to steady-state gas-exchange at each *PPFD*. Instead, *A*, *g*_*s*_, and *iWUE* showed substantial deviations from steady-state following changes in *PPFD*. Therefore, overall gas-exchange under fluctuating *PPFD* could be described as the sum of steady-state gas-exchange and the deviation of gas-exchange from steady-state following each *PPFD* fluctuation. To assess the importance of non-steady-state traits separate from steady-state, the deviation of gas-exchange from steady-state under fluctuating *PPFD* was calculated. Because steady-state and fluctuating *PPFD* response curves were measured on the same leaves, data from both types of curves were combined. Deviation of *A* from steady-state was calculated as follows: (1) *A* from the fluctuating *PPFD* response curves was normalized to *A*_2000_, (2) steady-state *A* from the steady-state *PPFD* response curve was obtained for each *PPFD*, and also normalized to *A*_2000_, (3) The difference between (1) and (2) was calculated at each *PPFD*, yielding the deviation of *A* from steady-state, normalized to *A*_2000_. This deviation is positive when *A* is greater than steady-state, negative when *A* is less than steady-state, and 0 when *A* is equal to steady-state. The average deviation of *A* from steady-state was calculated following increases and decreases in *PPFD*. The same method was applied to *iWUE*.

To test whether leaves cut from plants and measured in the laboratory might behave differently from leaves still attached to plants, an experiment was performed comparing the two types of leaves. There was no substantial effect of leaf excision on steady-state or non-steady-state *A*, *g*_*s*_, and *iWUE* (Supplementary Figure 9).

### Statistical Analysis

A summary of all traits analyzed in this study is given in Table 1. ANOVA was used to test the fixed effect of sorghum accession on gas-exchange and physiology traits with the aov() function in the package stats (R 3.6.1, R Core Team, 2017). There were 3–5 plants sampled per accession, with one plot per accession, such that each plant was a pseudo replicate. Time of day of measurements and leaf-to-air VPD were included as cofactors for gas-exchange measurements. If neither cofactor was significant for a given trait, then a simpler model testing only the effect of accession was used. *A*_2000_, *g_*s* 2000_*, and *iWUE*_2000_ were the average of two technical replicates per plant, and stomatal density and size were the average of four technical replicates per plant; all other measurements had one technical replicate per plant.

**TABLE 1:** Trait abbreviations and definitions.

**Trait**	**Abbreviation**	**Measurements used**
Steady-state *A* at *PPFD* = 2000 μmol m^–2^ s^–1^	*A*_2000_	Steady-state *PPFD* curve
Steady-state *g*_*s*_ at *PPFD* = 2000 μmol m^–2^ s^–1^	*g_*s* 2000_*	Steady-state *PPFD* curve
Steady-state *iWUE* at *PPFD* = 2000 μmol m^–2^ s^–1^	*iWUE*_2000_	Steady-state *PPFD* curve
Time required for *A* to come within 5% of steady-state after a decrease in *PPFD*	*t_95 A_*	Steady-state *PPFD* curve
Time required for *g*_*s*_ to come within 5% of steady-state after a decrease in *PPFD*	*t_95 gs_*	Steady-state *PPFD* curve
Time required for *iWUE* to come within 5% of steady-state after a decrease in *PPFD*	*t_95 iWUE_*	Steady-state *PPFD* curve
Undershoot of steady-state *A* after a decrease in *PPFD*	*A*_*undershoot*_	Steady-state *PPFD* curve
Undershoot of steady-state *g*_*s*_ after a decrease in *PPFD*	*g*_*s undershoot*_	Steady-state *PPFD* curve
Undershoot of steady-state *iWUE* after a decrease in *PPFD*	*iWUE*_*undershoot*_	Steady-state *PPFD* curve
Average *A* under fluctuating *PPFD*		Fluctuating *PPFD* curve
Average *iWUE* under fluctuating *PPFD*		Fluctuating *PPFD* curve
Average deviation of *A* from steady-state following increases in *PPFD*		Steady-state and fluctuating *PPFD* curves
Average deviation of *A* from steady-state following decreases in *PPFD*		Steady-state and fluctuating *PPFD* curves
Average deviation of *iWUE* from steady-state following increases in *PPFD*		Steady-state and fluctuating *PPFD* curves
Average deviation of *iWUE* from steady-state following decreases in *PPFD*		Steady-state and fluctuating *PPFD* curves
Stomatal density		Stomatal profiles
Stomatal size		Stomatal profiles

Homogeneity of variances was tested by the Levene test using function LeveneTest() in package DescTools (Signorell, 2020) and normality of studentized residuals tested by Shapiro–Wilk using function ols_test_normality in package olsrr (Hebbali, 2020) at *p* = 0.01 threshold. The assumption of normality was violated for *t_95 A_*, so the Kruskal–Wallis test was used to analyze this trait using kruskal.test() function in package stats (R Core Team, 2017).

Pairwise correlations were tested at *p* < 0.05 (significant) and *p* < 0.1 (marginally significant) thresholds between means per accession for the traits described above using cor.mtest() function in package corrplot (Wei and Simko, 2017).

## Results

### Variation Among Accessions in Steady-State Gas Exchange and in the Transition From One Steady-State to the Next Following Decreases in PPFD

**FIGURE 1:**
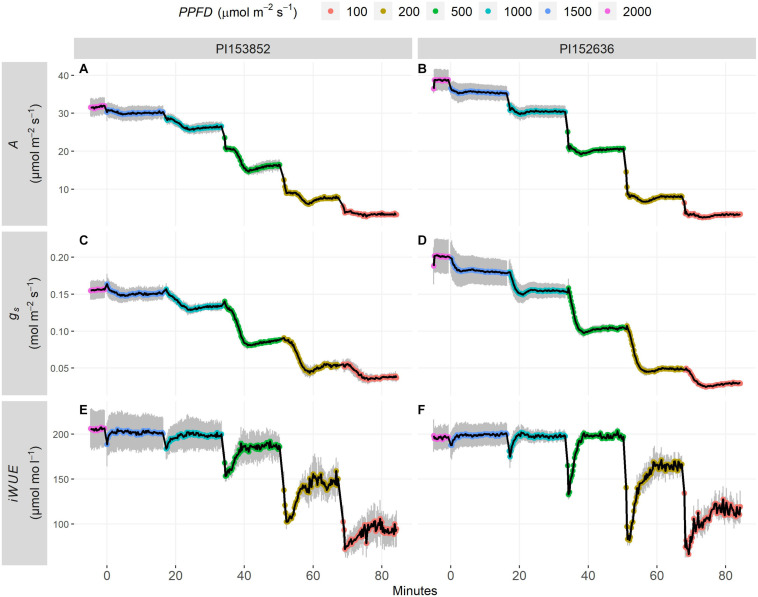
Timecourses of steady-state *PPFD* response curves in two accessions: **(A,C,E)** PI153852 and **(B,D,F)** PI152636. For each curve, leaves were acclimatized to *PPFD* of 2000 μmol m^–2^ s^–1^ for 1 h, then *PPFD* declined in steps every 15 min. **(A,B)** *A*, **(C,D)** *g*_*s*_, and **(E,F)** *iWUE* were logged throughout. Each point is a mean ± s.e. of 4 plants. The final measurements at each *PPFD* were used to estimate steady-state *A*, *g*_*s*_, and *iWUE* at each *PPFD*. Timecourses were also used to describe the transition from one steady-state to the next after each decrease in *PPFD*, including the time for *A*, *g*_*s*_, and *iWUE* to come within 5% of steady-state, and the undershoot of steady-state by *A*, *g*_*s*_, and *iWUE*.

Two representative accessions, PI153852 and PI152636, exemplify the genetic variation that was observed in steady-state and non-steady-state *A*, *g*_*s*_, and *iWUE* (Figure 1). In PI153852, steady-state *A* and *g*_*s*_ at *PPFD* = 2000 μmol m^–2^ s^–1^ were less than in PI152636 (i.e., *A*_2000_ and *g_*s* 2000_*, pink datapoints in Figures 1A–D, respectively). After each decrease in *PPFD*, *A* and *g*_*s*_ declined from one steady-state to the next. In PI153852, decline of *A* occurred over the course of several minutes, during which *A* decreased below steady-state (i.e., undershoot), then increased again to steady-state (Figure 1A). In contrast, in PI152636, *A* reached a new steady-state in under a minute, with a less pronounced undershoot (Figure 1B). In PI153852, *g*_*s*_ gradually decreased below steady-state (i.e., undershoot), then increased again to steady-state (Figure 1C). In contrast, in PI152636 *g*_*s*_ declined more rapidly to steady-state, with a slightly less pronounced undershoot (Figure 1D).

After each decrease in *PPFD*, *iWUE* abruptly declined below steady-state (i.e., undershoot), reflecting an instantaneous loss of *A* while *g*_*s*_ remained relatively high. This decline was less pronounced in PI153852 (Figure 1E) than PI152636 (Figure 1F). In the following minutes, *iWUE* increased to reach steady-state, reflecting a decline of *g*_*s*_ to steady-state. This occurred slightly more rapidly in PI153852 (Figure 1E) than in PI152636 (Figure 1F). This suggests that the differences between these two accessions in *A* and *g*_*s*_ at steady-state and non-steady-state translated to differences in *iWUE*. Steady-state *iWUE* was relatively stable from *PPFD* = 500–2000 μmol m^–2^ s^–1^, but began to decline at lower *PPFD* (Figures 1E,F and Supplementary Figure 1).

**FIGURE 2:**
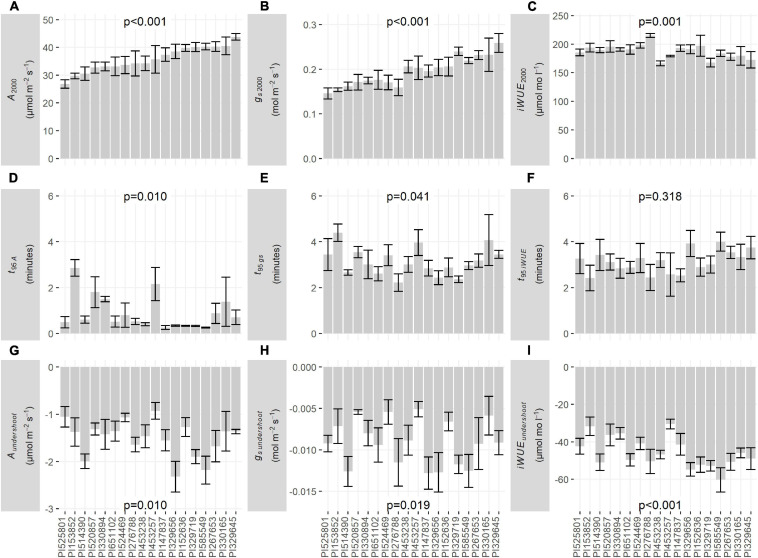
Bar graphs of traits derived from steady-state *PPFD* response curves: **(A)** steady-state *A* at *PPFD* = 2000 μmol m^–2^ s^–1^ (*A*_2000_), **(B)** steady-state *g*_*s*_ at *PPFD* = 2000 μmol m^–2^ s^–1^ (*g_*s* 2000_*), **(C)** steady-state *iWUE* at *PPFD* = 2000 μmol m^–2^ s^–1^ (*iWUE*_2000_), **(D)** time required for *A* to come within 5% of steady-state after a decrease in *PPFD* (*t_95 A_*), **(E)** time required for *g*_*s*_ to come within 5% of steady-state after a decrease in *PPFD* (*t_95 gs_*), **(F)** time required for *iWUE* to come within 5% of steady-state after a decrease in *PPFD* (*t_95 iWUE_*), **(G)** undershoot of steady-state by *A* after a decrease in *PPFD*, i.e., difference between steady-state *A* and the minimum *A* reached at each *PPFD* (*A*_*undershoot*_), **(H)** undershoot of steady-state by *g*_*s*_ after a decrease in *PPFD*, i.e., difference between steady-state *g*_*s*_ and the minimum *g*_*s*_ reached at each *PPFD* (*g*_*s*_ *_*undershoot*_*), **(I)** undershoot of steady-state by *iWUE* after a decrease in *PPFD*, i.e., difference between steady-state *iWUE* and the minimum *iWUE* reached at each *PPFD* (*iWUE*_*undershoot*_). Bars are mean ± s.e. *p*-values are from ANOVA testing the fixed effect of accession on each trait.

Among all 18 accessions, there was significant variation in *A*_2000_ (*p* < 0.001, 1.63-fold variation among accessions, Figure 2A), *g_*s* 2000_* (*p* < 0.001, 1.78-fold variation among accessions, Figure 2B), and *iWUE*_2000_ (*p* = 0.001, 1.29-fold variation among accessions, Figure 2C), as well as the time required for *A* (*t_95 A_*, *p* = 0.010, 11-fold variation among accessions, Figure 2D) and *g*_*s*_ (*t_95 gs_*, *p* = 0.041, 1.98-fold variation among accessions, Figure 2E) to come within 5% of steady-state after a decrease in *PPFD*. However, there was no significant variation in the time required for *iWUE* to come within 5% of steady-state after a decrease in *PPFD* (*t_95 iWUE_*, *p* = 0.318, 1.65-fold variation among accessions, Figure 2F). There was significant variation in the undershoot of steady-state by *A* (*A*_*undershoot*_, *p* = 0.010, 2.50-fold variation among accessions, Figure 2G), *g*_*s*_ (*g*_*s undershoot*_, *p* = 0.019, 2.51-fold variation among accessions, Figure 2H), and *iWUE* (*iWUE*_undershoot_, *p* < 0.001, 1.98-fold variation among accessions, Figure 2I) after each decrease in *PPFD*. *A* approached steady-state within seconds in most accessions (*t_95 A_* < 1 min, Figure 2D), with the slowest decline of *A* in PI153852 (*t_95 A_* = 2.86 min, Figures 1A, 2D). *g*_*s*_ and *iWUE* approached steady-state more slowly than *A* (*t_95 gs_* and *t_95 iWUE_* ranging from 2.22 to 4.40 min, Figures 2E,F).

### Variation Among Accessions in Gas Exchange Under Fluctuating PPFD

**FIGURE 3:**
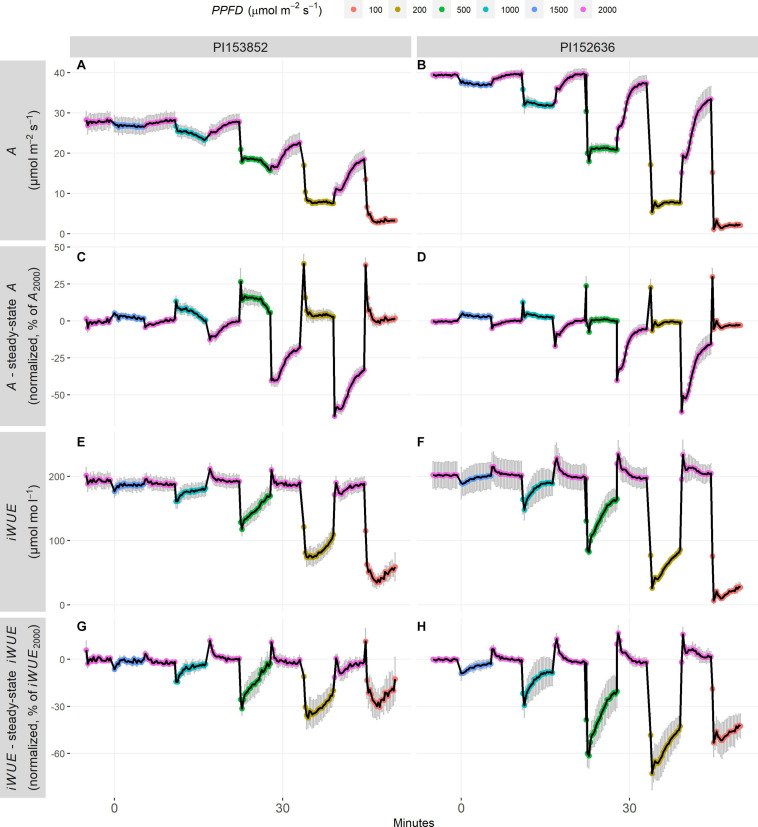
Timecourses of fluctuating *PPFD* response curves in two accessions: **(A,C,E,G)** PI153852 and **(B,D,F,H)** PI152636. For each curve, leaves were acclimatized to *PPFD* of 2000 μmol m^–2^ s^–1^ for 1 h, then *PPFD* cycled between non-saturating and saturating *PPFD* every 5.5 min. **(A,B)** *A*, **(C,D)** Deviation of *A* from steady-state, normalized to *A*_2000_, **(E,F)** *iWUE*, **(G,H)** Deviation of *iWUE* from steady-state, normalized to *iWUE*_2000_. Each point is a mean ± s.e. of 3–5 plants. Deviation of *A* from steady-state was calculated as follows: (1) *A* from a fluctuating *PPFD* response curve was normalized to *A*_2000_, (2) the steady-state *A* from a steady-state *PPFD* curve measured on the same leaf was obtained for each *PPFD*, and also normalized to *A*_2000_, (3) The difference between (1) and (2) was calculated, yielding the deviation of *A* from steady-state, normalized to *A*_2000_, as seen in panels **(C,D)**. This deviation is positive when *A* is greater than steady-state, negative when *A* is less than steady-state, and 0 when *A* is equal to steady-state. The same method was applied to *iWUE*, as seen in panels **(G,H)**.

Fluctuating *PPFD* response curves were used to determine whether the traits derived from steady-state *PPFD* response curves were predictive of performance in a fluctuating *PPFD* environment. Following decreases in *PPFD*, *A* rapidly declined to steady-state, while increases in *PPFD* triggered more gradual increases of *A* toward steady-state (Figure 3A,B). As a result, *A* was slightly above steady-state following decreases in *PPFD*, but substantially below steady-state following increases in *PPFD* (Figures 3C,D). As in the steady-state *PPFD* response curves, in accessions such as PI152636 there was little deviation of *A* from steady-state following decreases in *PPFD*, whereas in PI153852, *A* remained above steady-state for several minutes (Figures 3C,D).

Following decreases in *PPFD*, *iWUE* abruptly declined, then gradually increased toward steady-state (Figures 3E,F). In contrast, increases in *PPFD* caused *iWUE* to rise slightly above steady-state, then return to steady-state (Figures 3E,F). In other words, *iWUE* was slightly above steady-state following increases in *PPFD*, but substantially less than steady-state following decreases in *PPFD* (Figures 3G,H). This resulted from the fact that *A* declined faster than *g*_*s*_ following decreases in *PPFD*, whereas *A* and *g*_*s*_ increased at a similar rate following increases in *PPFD* (Supplementary Figures 5–7). In accessions such as PI152636, there was substantial loss of *iWUE* relative to steady-state following decreases in *PPFD*, and a slight gain of *iWUE* relative to steady-state following increases in *PPFD*, compared to the less pronounced deviation of *iWUE* from steady-state in accessions such as PI153852 (Figures 3G,H).

**FIGURE 4:**
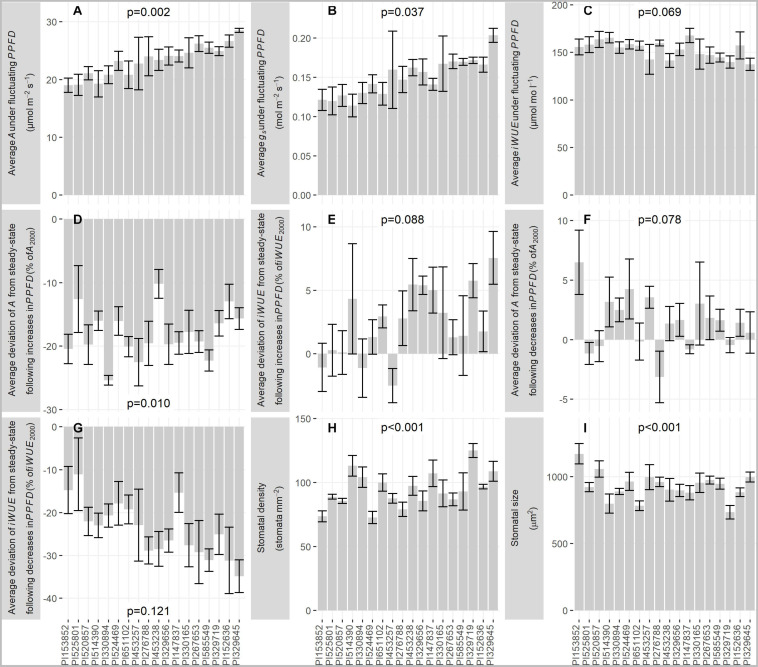
Bar graphs of traits derived from fluctuating *PPFD* response curves and leaf stomatal profiles: **(A)** Average *A* under fluctuating *PPFD*, **(B)** Average *g*_*s*_ under fluctuating *PPFD*, **(C)** Average *iWUE* under fluctuating *PPFD*, **(D)** Deviation of *A* from steady-state following increases in *PPFD*, expressed as a% of *A*_2000_, **(E)** Deviation of *iWUE* from steady-state following increases in *PPFD*, expressed as a% of *iWUE*_2000_, **(F)** Deviation of *A* from steady-state following decreases in *PPFD*, expressed as a% of *A*_2000_, **(G)** Deviation of *iWUE* from steady-state following decreases in *PPFD*, expressed as a% of *iWUE*_2000_, **(H)** stomatal density, **(I)** stomatal size, i.e., planar surface area of the stomatal complex. Bars are mean ± s.e. *p*-values are from ANOVA testing the fixed effect of accession on each trait.

**FIGURE 5:**
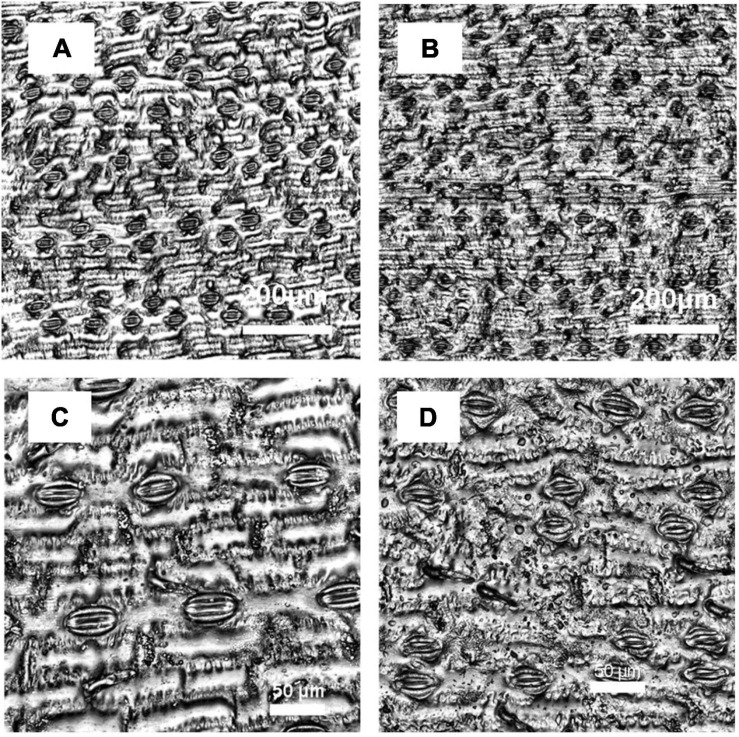
Optical topometry images of leaf abaxial surfaces used for stomatal profiles, measured with: **(A,B)**, 20× magnification, **(C,D)**, 50× magnification. Panels **(A,C)** show representative examples for an accession (PI524469) with low stomatal density (73 stomata mm^–2^) and large stomata (964 μm^2^), and **(B,D)** for an accession (PI329719) with high stomatal density (125 stomata mm^–2^) and small stomata (734 μm^2^).

Among all accessions, there was significant or marginally significant variation in average *A* under fluctuating *PPFD* (*p* = 0.002, 1.50-fold variation among accessions, Figure 4A), average *g*_*s*_ under fluctuating *PPFD* (*p* = 0.037, 1.78-fold variation among accessions, Figure 4B), and average *iWUE* under fluctuating *PPFD* (*p* = 0.069, 1.22-fold variation among accessions, Figure 4C), as well as the deviation of *A* from steady-state following increases in *PPFD* (*p* = 0.010, 2.50-fold variation among accessions, Figure 4D), the deviation of *iWUE* from steady-state following increases in *PPFD* (*p* = 0.088, 4.02-fold variation among accessions, Figure 4E), and the deviation of *A* from steady-state following decreases in *PPFD* (*p* = 0.078, 3.07-fold variation among accessions, Figure 4F). However, the experiment could not resolve significant differences among accessions for the deviation of *iWUE* from steady-state following decreases in *PPFD* (*p* = 0.121, 3.15-fold variation among accessions, Figure 4G). Finally, there was significant variation among accessions in stomatal density (*p* < 0.001, 1.72-fold variation among accessions, Figure 4H) and stomatal size (*p* < 0.001, 1.59-fold variation among accessions, Figure 4I) determined from optical topometry of the epidermis (Figure 5).

### Correlations Between A, g_*s*_, iWUE, and Stomatal Patterning Traits

#### Traits Correlated With Steady-State A, g_*s*_, and iWUE

**FIGURE 6:**
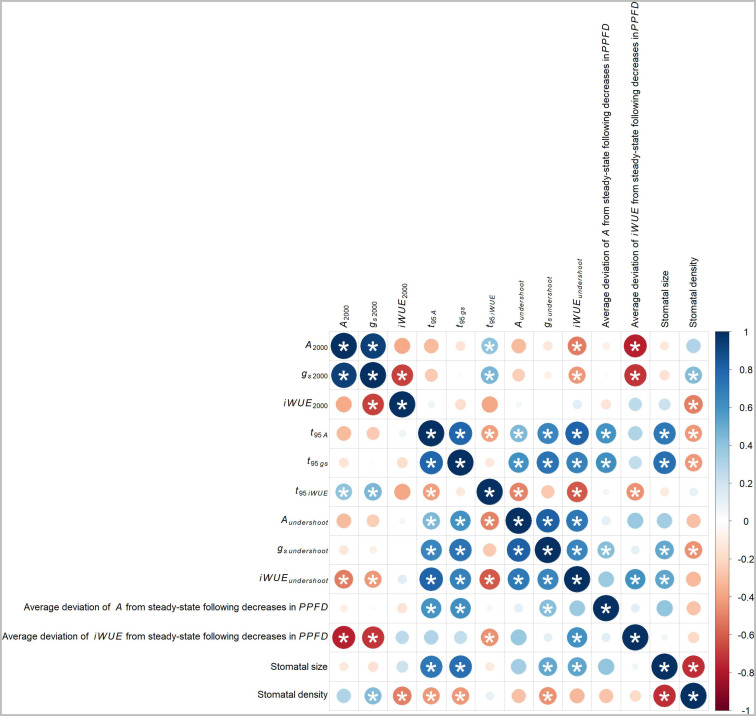
Pearson’s correlation coefficients (*r*) for traits potentially underlying variation in non-steady-state *iWUE* following decreases in *PPFD*. The size of circles gives the strength of correlation and the color gives the direction of correlation: red (negative) and blue (positive). Significance (*p* < 0.1 threshold) is marked by *. The corresponding pairwise correlation scatterplots are given in Supplementary Figure 8.

Steady-state *g_*s* 2000_* was positively correlated with *A*_2000_ (*p* < 0.001, *R*^2^ = 0.87, Figure 6) and negatively correlated with *iWUE*_2000_ (*p* = 0.0019, *R*^2^ = 0.46, Figure 6). After a decrease in *PPFD*, *iWUE* increased toward steady-state more slowly in accessions with greater *A*_2000_ and *g_*s* 2000_* (positive correlation of *A*_2000_ with *t_95 iWUE_*, *p* = 0.094, *R*^2^ = 0.17; positive correlation of *g_*s* 2000_* with *t_95 iWUE_*, *p* = 0.059, *R*^2^ = 0.2, Figure 6). After a decrease in *PPFD*, undershoot of steady-state *iWUE* was more pronounced in accessions with greater *A*_2000_ and *g_*s* 2000_* (negative correlation of *A*_2000_ with *iWUE_*undershoot*_*, *p* = 0.03, *R*^2^ = 0.26; negative correlation of *g_*s* 2000_* with *iWUE*_*undershoot*_, *p* = 0.074, *R*^2^ = 0.19, Figure 6). Accordingly, deviation of *iWUE* from steady-state following decreases in *PPFD* was more negative in accessions with greater *A*_2000_ (*p* < 0.001, *R*^2^ = 0.61, Figure 6) and greater *g_*s* 2000_* (*p* < 0.001, *R*^2^ = 0.52, Figure 6).

#### Traits Correlated With Non-Steady-State A

For non-steady-state *A* to be maximized following a decrease in *PPFD*, *A* should slowly approach steady-state (i.e., high *t_95 A_*) with minimal undershoot (i.e., greater, i.e., less negative, *A*_*undershoot*_). This non-steady-state *A* may also be related to non-steady-state *g*_*s*_. Accordingly, accessions with a more positive deviation of *A* from steady-state following decreases in *PPFD*, also had greater *t_95 A_* (*p* = 0.0088, *R*^2^ = 0.36, Figure 6), greater *t_95 gs_* (*p* = 0.0062, *R*^2^ = 0.38, Figure 6), and less negative *g_*s undershoot*_* (*p* = 0.078, *R*^2^ = 0.18, Figure 6). In accessions in which *A* and *g*_*s*_ approached steady-state more slowly (i.e., greater *t_95 A_* and *t_95 gs_*), the undershoot of steady-state *A* and *g*_*s*_ was less pronounced (i.e., less negative *A*_*undershoot*_ and *g*_*s undershoot*_). This was evidenced by the positive correlation of *t_95 A_* with *A*_*undershoot*_ (*p* = 0.063, *R*^2^ = 0.2, Figure 6) and *g*_*s undershoot*_ (*p* = 0.0033, *R*^2^ = 0.43, Figure 6) and the positive correlation of *t_95 gs_* with *A*_*undershoot*_ (*p* = 0.0081, *R*^2^ = 0.36, Figure 6) and *g*_*s undershoot*_ (*p* < 0.001, *R*^2^ = 0.54, Figure 6).

Non-steady-state *A* and *g*_*s*_ were coordinated following a decrease in *PPFD*. In accessions in which *A* approached steady-state more slowly (i.e., greater *t_95 A_*) with a less pronounced undershoot (i.e., less negative *A*_*undershoot*_), the same was also seen for *g*_*s*_ (i.e., greater *t_95 gs_* and less negative *g*_*s undershoot*_). This was evidenced by the positive correlation of *t_95 A_* with *t_95 gs_* (*p* < 0.001, *R*^2^ = 0.63, Figure 6) and the positive correlation of *A*_*undershoot*_ with *g*_*s undershoot*_ (*p* < 0.001, *R*^2^ = 0.66, Figure 6).

#### Traits Correlated With Non-Steady-State iWUE

For non-steady-state *iWUE* to be maximized following a decrease in *PPFD*, *iWUE* should rapidly approach steady-state (i.e., small *t_95*iWUE*_*) with minimal undershoot (i.e., greater, i.e., less negative *iWUE*_*undershoot*_). Accordingly, accessions with a less negative deviation of *iWUE* from steady-state following decreases in *PPFD* also had smaller *t_95*iWUE*_* (*p* = 0.051, *R*^2^ = 0.21, Figure 6), and greater *iWUE_*undershoot*_* (*p* = 0.0074, *R*^2^ = 0.37, Figure 6).

Non-steady-state *iWUE* traits were associated with *A* rather than *g*_*s*_. Specifically, in accessions in which *iWUE* approached steady-state more slowly (i.e., greater *t_95*iWUE*_*), *A* approached steady-state more rapidly with a more pronounced undershoot (i.e., smaller *t_95 A_* and more negative *A*_*undershoot*_). This was evidenced by the negative correlation of *t_95*iWUE*_* with *t_95 A_* (*p* = 0.085, *R*^2^ = 0.17, Figure 6) and *A*_*undershoot*_ (*p* = 0.035, *R*^2^ = 0.25, Figure 6). Additionally, accessions which displayed a less pronounced undershoot of steady-state by *iWUE* following decreases in *PPFD* (i.e., greater, i.e., less negative *iWUE*_*undershoot*_) also had a slower decrease of *A* and *g*_*s*_ to reach steady-state (positive correlation of *iWUE*_*undershoot*_ with *t_95 A_*, *p* < 0.001, *R*^2^ = 0.65; positive correlation of *iWUE*_*undershoot*_ with *t_95 gs_*, *p* = 0.0021, *R*^2^ = 0.46, Figure 6). Further, in accessions which displayed a less pronounced undershoot of steady-state by *iWUE*, the same was seen for *A* and *g*_*s*_ (positive correlation of *iWUE*_*undershoot*_ with *A*_*undershoot*_, *p* < 0.001, *R*^2^ = 0.51; positive correlation of *iWUE*_*undershoot*_ with *g*_*s undershoot*_, *p* = 0.0034, *R*^2^ = 0.42, Figure 6). Finally, accessions in which *iWUE* approached steady-state more slowly (i.e., greater *t_95 iWUE_*) had a more pronounced undershoot of steady-state *iWUE* (negative correlation of *t_95 iWUE_* with *iWUE*_*undershoot*_, *p* = 0.0068, *R*^2^ = 0.38, Figure 6).

These surprising findings are exemplified in accessions PI153852 and PI152636 (Figure 1). PI152636 displayed faster declines in *g*_*s*_ after each decrease in *PPFD* (i.e., smaller *t_95 gs_*, Figure 1D), which theoretically would lead to increased *iWUE*, when compared to the slower *g*_*s*_ response of PI153852 (Figure 1C). However, in PI153852 the slower response of *g*_*s*_ was also associated with slower, more gradual declines in *A* after each decrease in *PPFD* (i.e., greater *t_95 A_*, Figure 1A), which would also theoretically lead to increased *iWUE*, when compared to the faster *A* response of PI152636 (Figure 1B). The net result in terms of *iWUE* yielded a benefit to PI153852 (Figure 1E), with a less pronounced undershoot of steady-state *iWUE* (i.e., less negative *iWUE*_*undershoot*_) and a faster return to steady-state *iWUE* (i.e., smaller *t_95 iWUE_*) when compared to PI152636 (Figure 1F). In other words, when comparing PI153852, with slow responses of *A* and *g*_*s*_ to decreases in *PPFD*, to PI152636, with fast responses of *A* and *g*_*s*_ to decreases in *PPFD*, PI153852 showed the greatest *iWUE* at non-steady-state.

#### Correlations With Stomatal Density and Size

Stomatal density and size were negatively correlated (*p* < 0.001, *R*^2^ = 0.54, Figure 6). Accessions with more numerous, smaller stomata had greater steady-state *g*_*s*_ and more rapid responses of *A* and *g*_*s*_ to decreases in *PPFD*. Specifically, accessions with greater stomatal density had greater *g_*s* 2000_* (*p* = 0.074, *R*^2^ = 0.19, Figure 6), lower *iWUE*_2000_ (*p* = 0.032, *R*^2^ = 0.26, Figure 6), smaller *t_95 A_* (*p* = 0.073, *R*^2^ = 0.19, Figure 6), smaller *t_95 gs_* (*p* = 0.069, *R*^2^ = 0.19, Figure 6), and more negative *g*_*s undershoot*_ (*p* = 0.059, *R*^2^ = 0.21, Figure 6). Accessions with smaller stomata had smaller *t_95 A_* (*p* < 0.001, *R*^2^ = 0.51, Figure 6), smaller *t_95 gs_* (*p* < 0.001, *R*^2^ = 0.56, Figure 6), more negative *g*_*s undershoot*_ (*p* = 0.029, *R*^2^ = 0.26, Figure 6), and more negative *iWUE*_undershoot_ (*p* = 0.024, *R*^2^ = 0.28, Figure 6).

#### Overall A and iWUE Under Fluctuating PPFD: Contributions of Steady-State and Non-Steady-State Gas-Exchange

**FIGURE 7:**
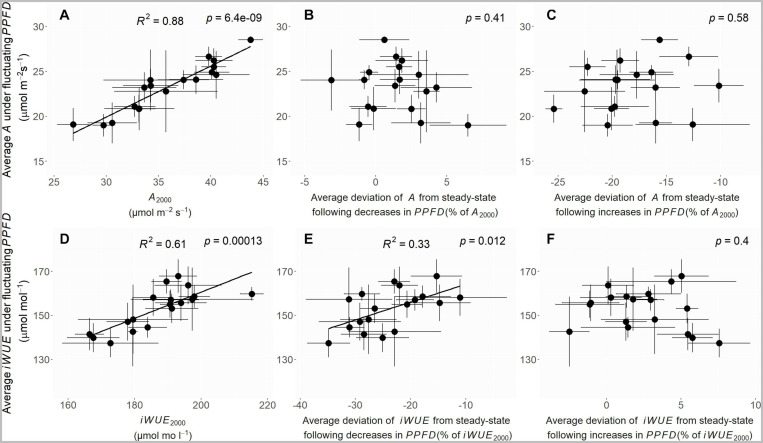
Correlation plots of average *A* under fluctuating *PPFD* against: **(A)** *A*_2000_, **(B)** the average deviation of *A* from steady-state following decreases in *PPFD*, expressed as a% of *A*_2000_, **(C)** the average deviation of *A* from steady-state following increases in *PPFD*, expressed as a% of *A*_2000_; and correlation plots of average *iWUE* under fluctuating *PPFD* against: **(D)** *iWUE*_2000_, **(E)** the average deviation of *iWUE* from steady-state following decreases in *PPFD*, expressed as a% of *iWUE*_2000_, **(F)** the average deviation of *iWUE* from steady-state following increases in *PPFD*, expressed as a% of *iWUE*_2000_.

Overall gas-exchange under fluctuating *PPFD* could be described as the sum of steady-state gas-exchange and the deviation of gas-exchange from steady-state following each *PPFD* fluctuation. An additional correlation analysis was used to explore whether steady-state and non-steady-state gas-exchange were significantly associated with overall gas-exchange under fluctuating *PPFD*. Average *A* and *iWUE* under fluctuating *PPFD* were tested for correlation with steady-state *A*_2000_ and *iWUE*_2000_, and with the deviation of *A* and *iWUE* from steady-state following decreases and increases in *PPFD*. The majority of variation in average *A* under fluctuating *PPFD* was associated with variation in steady-state *A*_2000_ (*p* < 0.001, *R*^2^ = 0.88, Figure 7A) but not with deviation of *A* from steady-state following decreases (*p* = 0.41, Figure 7B) or increases (*p* = 0.58, Figure 7C) in *PPFD*. The majority of variation in average *iWUE* under fluctuating *PPFD* was associated with variation in steady-state *iWUE*_2000_ (*p* < 0.001, *R*^2^ = 0.61, Figure 7D) and there was also substantial variation associated with deviation of *iWUE* from steady-state following decreases (*p* = 0.012, *R*^2^ = 0.33, Figure 7E), but not increases (*p* = 0.4, Figure 7F), in *PPFD*. This suggests a potentially important role for non-steady-state *iWUE* following decreases in *PPFD*, causing a substantial loss of *iWUE* under fluctuating *PPFD*.

## Discussion

Improving *iWUE* under fluctuating *PPFD* is important to sustain or further increase crop yields (Leakey et al., 2019). This study shows significant variation among sorghum accessions in steady-state and non-steady-state *A* and *g*_*s*_ and stomatal density and size, reveals how variation in these traits drives variation in *iWUE*, and discusses how tradeoffs between these should shape strategies for decreasing crop water use.

### Decreases in PPFD Substantially Impair iWUE Under Fluctuating PPFD, With Loss of Non-Steady-State iWUE Associated With A Rather Than g_*s*_

Almost all variation in average *A* under fluctuating *PPFD* was associated with steady-state *A*_2000_ (Figures 7A–C). In contrast, much of the variation in average *iWUE* under fluctuating *PPFD* was associated with the non-steady-state loss of *iWUE* following decreases in *PPFD* in addition to steady-state *iWUE*_2000_ (Figures 7D–F). This points to non-steady-state *iWUE* as an important contributor to overall *iWUE* under fluctuating *PPFD*, with decreases in *PPFD* being more impactful than increases in *PPFD*. This could in large part be attributed to the undershoot of steady-state by *iWUE* following decreases in *PPFD* (i.e., *iWUE*_*undershoot*_), which showed significant variation among accessions and so could be a promising target for improvement.

The response of *iWUE* to a decrease in *PPFD* was biphasic, with an abrupt loss of *iWUE* driven by *A*, followed by a gradual return to steady-state driven by *g*_*s*_ (Figure 1). Loss of *iWUE* was mitigated in accessions in which the undershoot of steady-state *iWUE* was less pronounced (i.e., greater, i.e., less negative, *iWUE*_*undershoot*_) and *iWUE* returned to steady-state more rapidly (i.e., smaller *t_95 iWUE_*, Figure 6). The finding that *t_95 iWUE_* and *iWUE*_*undershoot*_ were associated with corresponding traits of *A* rather than *g*_*s*_ is novel, and suggests that breeding for improved non-steady-state *A* traits, i.e., high *t_95 A_* and less negative *A*_*undershoot*_, could mitigate loss of *iWUE* under fluctuating *PPFD* (Figure 6).

Non-steady-state *A* following decreases in *PPFD* is influenced by different physiological processes. Undershoot of steady-state by *A* following decreases in *PPFD* may be attributed to kinetics of protective energy-dissipating mechanisms, collectively termed non-photochemical quenching, and photorespiration (Kaiser et al., 2015, 2018), and has been observed in C_3_ dicots such as Arabidopsis, French bean (McAusland et al., 2016) and tobacco (Kromdijk et al., 2016). This could explain the rapid and pronounced undershoot of steady-state *A* following decreases in *PPFD* in accessions such as PI329656 (Supplementary Figure 2G). Large pools of metabolites involved in C_4_ photosynthesis could buffer energy supply and sustain a higher *A* for some time following a decrease in *PPFD* (Stitt and Zhu, 2014). For instance, a large pool of active malate carries enough reductive power to reduce CO_2_ for several seconds after a light to dark transition (Slattery et al., 2018). C_4_ activity could be insufficient to achieve CO_2_ saturation of Rubisco and eliminate photorespiration at low *PPFD*, leading to reduced steady-state *A* (Kromdijk et al., 2010). If so, a leaf with CO_2_-saturated Rubisco at high *PPFD* could maintain CO_2_-saturation for some time after a decrease in *PPFD*, effectively maintaining photorespiration below its steady-state and therefore boosting *A*. This could explain the relatively gradual decline of *A* following decreases in *PPFD* in accessions such as PI153852 (Figure 1A), enabling it to maintain *A* above steady-state for several minutes following decreases in *PPFD* (Figure 3C). Here, we show that potential for improvement of non-steady-state *A* in sorghum through breeding is supported by significant variation in *t_95 A_* and *A*_*undershoot*_, which may result from variation in the processes above (Figure 2). This highlights the value of the methodology used in the present study to assess steady-state and non-steady-state gas-exchange traits, and demonstrates that sorghum is a relevant crop species to study diversity in these traits.

### Interactions Between Non-Steady-State A and g_*s*_ Impair iWUE Following Decreases in PPFD: This Could Be Resolved in Leaves With Smaller, More Sensitive Stomata

Many efforts to improve *iWUE* under fluctuating light have focused on faster stomatal closure (Lawson and Blatt, 2014; Bellasio et al., 2017; Lawson and Vialet-Chabrand, 2019). In a simplified model of leaf gas-exchange following a decrease in *PPFD*, where *A* has an instant step-change from one steady-state to the next, a faster decrease in *g*_*s*_ would directly lead to a faster increase in *iWUE*, leading to overall improvement in *iWUE* under fluctuating *PPFD*. In the sorghum accessions studied here, this process was complicated by interactions between *g*_*s*_ and *A*, which may reflect precise stomatal sensing of *A* (reviewed: Lawson and Matthews, 2020). In other words, accessions with rapid decreases in *g*_*s*_ also had rapid decreases in *A*, negating much of the benefit to *iWUE* (Figures 1, 6). In fact, the undershoot of steady-state *iWUE* following decreases in *PPFD* (i.e., *iWUE*_*undershoot*_) was most negative in accessions with faster stomatal responses (i.e., smaller *t_95 gs_*, Figure 6). If the same coordination between non-steady state *A* and *g*_*s*_ applies across species, this could explain the observation that faster stomatal closing speed did not translate to improved water saving across diverse plant species (Deans et al., 2019).

Because of this tradeoff, fast *g*_*s*_ response to decreasing *PPFD* may be a difficult target for improvement of *iWUE* through breeding in sorghum, though it may be possible to bypass this tradeoff through transgenic means. The optimal leaf response following decreases in *PPFD* would be a slow decline in *A* with minimal undershoot of steady-state, paired with a rapid decline in *g*_*s*_. This might be achieved in leaves with enhanced stomatal sensitivity to *A*, in which even a slow decline in *A* following a decrease in *PPFD* could trigger a rapid stomatal response. In particular, stomatal aperture responds to light via two separate pathways: the photosynthesis-independent and guard-cell specific blue light pathway, and the photosynthesis-dependent red light pathway. The latter is thought to be the main mechanism coordinating stomatal behavior with photosynthesis (Matthews et al., 2020). Therefore, manipulation of components involved in red light sensing, such as the redox state of the chloroplastic plastoquinone pool (Głowacka et al., 2018), could be a good target for manipulation to increase stomatal sensitivity to changes in *A* and improve coordination of *A* and *g*_*s*_.

Another factor influencing the speed of change in *g*_*s*_ is stomatal size, with smaller stomata generally showing faster movement, possibly due to greater guard cell membrane surface area to volume ratio (Drake et al., 2013; Raven, 2014; Lawson and Vialet-Chabrand, 2019; Lawson and Matthews, 2020). Therefore, leaves with smaller stomata might allow greater sensitivity of *g*_*s*_ to *A* by enabling mechanically faster stomatal closure. However, stomatal size is usually negatively correlated with stomatal density (Hetherington and Woodward, 2003). Here, stomatal density and size were negatively correlated (Figure 6), and leaves with more numerous and smaller stomata had faster *t_95 gs_* but also had reduced *iWUE*_2000_, more negative *iWUE*_*undershoot*_, and increased *g_*s* 2000_* (Figures 5, 6). This points to a tradeoff between leaves with more numerous, smaller stomata which show high steady-state *g*_*s*_ but rapid *g*_*s*_ responses to *PPFD*, and leaves with fewer, larger stomata which show low steady-state *g*_*s*_ but slow *g*_*s*_ responses to *PPFD*. The finding that steady-state *iWUE*_2000_ was mainly associated with *g_*s* 2000_* rather than *A*_2000_ is consistent with prior observations in sorghum (Geetika et al., 2019) and other C_4_ grasses (Leakey et al., 2019), but the tradeoff with non-steady-state *g*_*s*_ identified here (Figure 6) is not widely recognized.

Sorghum leaves with fewer and smaller stomata might achieve the best of both worlds with low steady-state *g*_*s*_ but rapid stomatal responses to *PPFD*. In C_4_ crops such as sorghum, where photosynthesis is typically CO_2_-saturated even under sub-ambient conditions, modestly reducing *g*_*s*_ at steady-state may not impair *A*, compounding benefits to *iWUE* (Leakey et al., 2019; Pignon and Long, 2020). Further, at a given *SD*, C_4_ grass stomata are smaller than those of related C_3_ grasses (Taylor et al., 2012). Transgenic approaches may hold potential to break the relationship between stomatal density and size (reviewed: Leakey et al., 2019).

Increases in *PPFD* were much less disruptive to *iWUE* than decreases in *PPFD* (Figure 3). The fact that *iWUE* was slightly above steady-state following increases in *PPFD* suggests that increase in *A* was faster than *g*_*s*_: this is in agreement with findings from a C_4_ stomata model (Bellasio et al., 2017) applied to photosynthesis induction data in maize (Chen et al., 2013). By comparison, in many other C_3_ and C_4_ dicots and monocots, the return of *iWUE* to steady-state following an increase in *PPFD* was much slower (e.g., >30 min), reflecting a pronounced desynchronization between *A* and *g*_*s*_ (McAusland et al., 2016). In our study, the response of *g*_*s*_ to an increase in *PPFD* occurred within seconds, whereas in C_3_ species there may be a lag of up to several minutes before stomata begin to open (Lawson and Blatt, 2014). Together these findings suggest exceptional coordination of *A* and *g*_*s*_ following increases in *PPFD* in sorghum.

### Relative to Other Species, iWUE in Sorghum Is High Both at Steady-State and Non-Steady-State

The range of natural variation among sorghum accessions tested here in *iWUE*_2000_ of 166–215 μmol mol^–1^ (Figure 2) is similar to published variation in sorghum RILs (100–140 μmol mol^–1^; Kapanigowda et al., 2014) and accessions (143–176 μmol mol^–1^; Xin et al., 2009), but less variable compared to measurements in closely related NADP-ME C_4_ grasses such as maize (80–140 μmol mol^–1^; Yabiku and Ueno, 2017), sugarcane (100–180 μmol mol^–1^; Viswanathan et al., 2014) and elephant grass (100–160 μmol mol^–1^; Sollenberger et al., 2014), whereas the widest range of variation tends to be found in C_3_ species such as soybean (40–115 μmol mol^–1^; Tomeo and Rosenthal, 2017), wheat (25–65 μmol mol^–1^; Jahan et al., 2014), and rice (50–80 μmol mol^–1^; Giuliani et al., 2013).

For both steady-state and non-steady-state traits, variation in *iWUE* was narrower than for *A* and *g*_*s*_ (Figures 2, 4). This resulted from coordination in *A* and *g*_*s*_, e.g., accessions with high *A*_2000_ also had high *g_*s* 2000_* and accessions with high *t_95 A_* also had high *t_95 gs_* (Figure 6). The accessions studied here showed faster *g*_*s*_ responses to changes in *PPFD* and higher *iWUE* compared to diverse gymnosperms and C_3_ dicots (Deans et al., 2019), C_3_ monocot crops such as wheat and rice, and even closely related C_4_ monocots such as maize and Miscanthus (Chen et al., 2013; McAusland et al., 2016). Understanding how sorghum maintains coordination between *A* and *g*_*s*_ to sustain high *iWUE* may be valuable to design strategies for improvement in species where coordination is less tight.

In our study, *g*_*s*_ declined over the course of the fluctuating *PPFD* timecourse, suggesting that stomatal opening during increases in *PPFD* was slower than stomatal closing during decreases in *PPFD* (Figure 3). Faster stomatal closure than opening may be a consequence of sorghum’s adaptation to dry, high-light environments, where water is more limiting than light and rapid stomatal closing can maximize *iWUE* (Vico et al., 2011; McAusland et al., 2016). On the contrary, species adapted to shaded environments such as a forest understory, where light is more limiting than water, typically show faster stomatal opening than closing, which can maximize *A* with little penalty to *iWUE*: these patterns suggest that dynamic stomatal traits are driven by ecological adaptation rather than evolutionary lineage (Deans et al., 2019). Increased steady-state *iWUE* of C_4_ photosynthesis may have been a driver for evolution of this pathway (Osborne and Sack, 2012). The fact that C_4_ grasses such as sorghum display fast stomatal responses to fluctuating light (Grantz and Assmann, 1991; Knapp, 1993; McAusland et al., 2016) may be an additional evolved mechanism to improve non-steady-state *iWUE*.

While a drought treatment was not included, we show genetic variation that may be exploited to reduce crop water demand and avoid drought (Leakey et al., 2019). An important next step will be to determine whether the trait correlations identified here are also observed in water-limited plants. Plants that develop under water-limited conditions can produce fewer leaves with fewer and/or smaller stomata to mitigate water loss. In these smaller plants, reduced canopy density may limit the prevalence of light fluctuations and alter the microenvironment including VPD and temperature. However, leaves that develop with sufficient water supply but are water-limited afterward have fewer options to acclimate. With stomatal density and size already fixed during development, stomatal closure is the main mechanism available to reduce steady-state *g*_*s*_. Leaves that permanently operate in a reduced range of stomatal apertures could have an altered relationship between steady-state traits (e.g., *g_*s* 2000_*) and non-steady-state traits (e.g., *t_95 gs_*, *g*_*s undershoot*_). This could also affect the relative association of *A* and *g*_*s*_ traits with *iWUE*: at low apertures, stomatal control may be less precise (Kaiser and Kappen, 2001) and so more wasteful in terms of water loss. An important next step will be to determine the degree of plasticity in steady-state and non-steady-state *A*, *g*_*s*_ and *iWUE* traits under different environments.

## Conclusion

In this study, we show that a common measurement, the steady-state *PPFD* response curve, can be used to derive valuable insight into both steady-state and non-steady-state *A*, *g*_*s*_ and *iWUE* (Figures 1, 2). The relevance of these non-steady-state traits could be seen when leaves were exposed to a fluctuating *PPFD* regime, as natural diversity in traits such as *t_95 A_*, *A*_*undershoot*_, and *iWUE*_*undershoot*_ correlated with the deviation of *A* and *iWUE* from steady-state under fluctuating *PPFD* (Figures 3, 4, 6). Remarkably, the deviation of *A* and *iWUE* from steady-state under fluctuating *PPFD* was substantial even under the relatively lengthy *PPFD* fluctuations, spaced 5.5 min apart, of the fluctuating *PPFD* response curves used here (Figure 7). In a crop canopy, where most light fluctuations are more rapid (<5 s) (Kaiser et al., 2018), the non-steady-state processes quantified here, especially the photosynthetic traits *t_95 A_* and *A*_*undershoot*_, would likely be even more important in driving overall *iWUE*. Variation among accessions in steady-state and non-steady-state traits may be exploited to reduce crop water demand and avoid drought, but our results emphasize that translating this into breeding strategies will require careful consideration of emerging tradeoffs due to co-variation between traits.

## Data Availability Statement

The original contributions presented in the study are included in the article/Supplementary Material, further inquiries can be directed to the corresponding author/s.

## Author Contributions

CP designed and performed the research, data collection, analysis and interpretation, and wrote the manuscript. AL, SL, and JK performed the data analysis and interpretation and wrote the manuscript. All authors contributed to the article and approved the submitted version.

## Conflict of Interest

The authors declare that the research was conducted in the absence of any commercial or financial relationships that could be construed as a potential conflict of interest.

## Supplementary Material

Supplementary Figure 1 Steady-state *PPFD* response curves for *A*, *g*_*s*_, and *iWUE* in all accessions.

Supplementary Figure 2 Timecourses of steady-state *A*-*PPFD* response curves in all accessions.

Supplementary Figure 3 Timecourses of steady-state *g_*s*_-PPFD* response curves in all accessions.

Supplementary Figure 4 Timecourses of steady-state *iWUE-PPFD* response curves in all accessions.

Supplementary Figure 5 Timecourses of fluctuating *PPFD* response curves for *A* in all accessions.

Supplementary Figure 6 Timecourses of fluctuating *PPFD* response curves for *g*_*s*_ in all accessions.

Supplementary Figure 7 Timecourses of fluctuating *PPFD* response curves for *iWUE* in all accessions.

Supplementary Figure 8 Pearson’s correlation coefficients, pairwise correlation scatterplots, and density plots for traits potentially underlying variation in non-steady-state *iWUE* following decreases in *PPFD*.

Supplementary Figure 9 Test of the effect of leaf excision on steady-state and non-steady-state leaf gas-exchange.

Supplementary Table 1 Variation in environmental controls during gas-exchange measurements.
