# Supplementary material for: Drivers of Natural Variation in Water-Use Efficiency Under Fluctuating Light Are Promising Targets for Improvement in Sorghum
Source: Front Plant Sci. 2021 Feb 1;12:627432. doi: 10.3389/fpls.2021.627432 (PMC7882533; doi:10.3389/fpls.2021.627432)
Supplement: Supplementary Figure 1 — Steady-state PPFD response curves for A, gs, and iWUE in all accessions. [file Data_Sheet_1.docx]

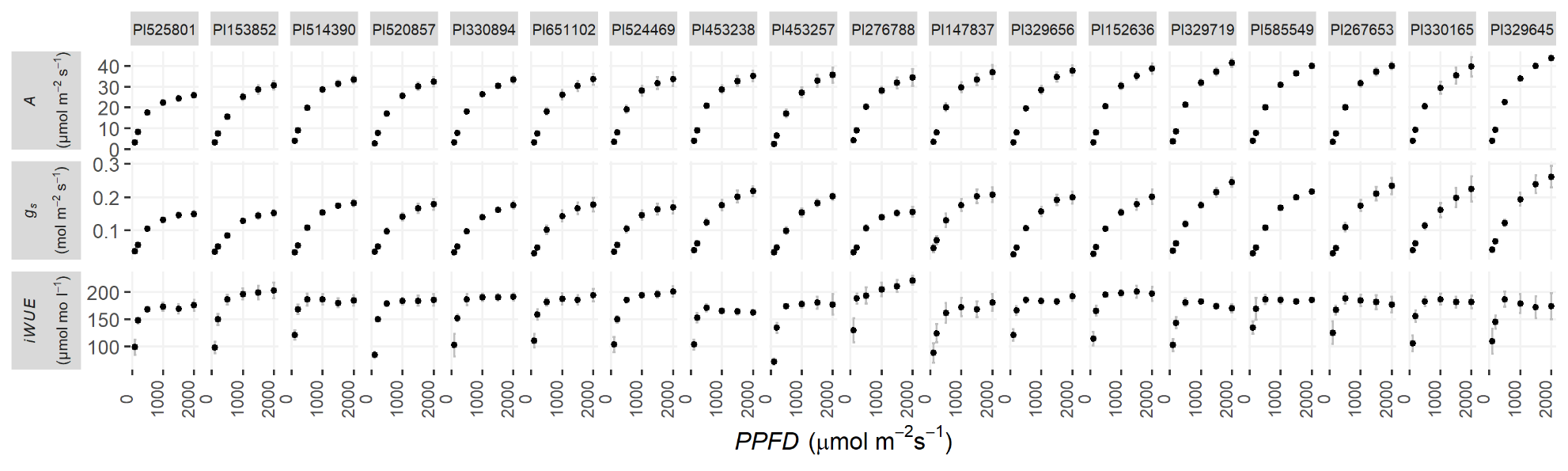


Supplementary Fig. S1: Steady-state *PPFD* response curves for *A*, *g_s_* and *iWUE* in all accessions. Each point is a mean ± s.e. of 3-5 plants.


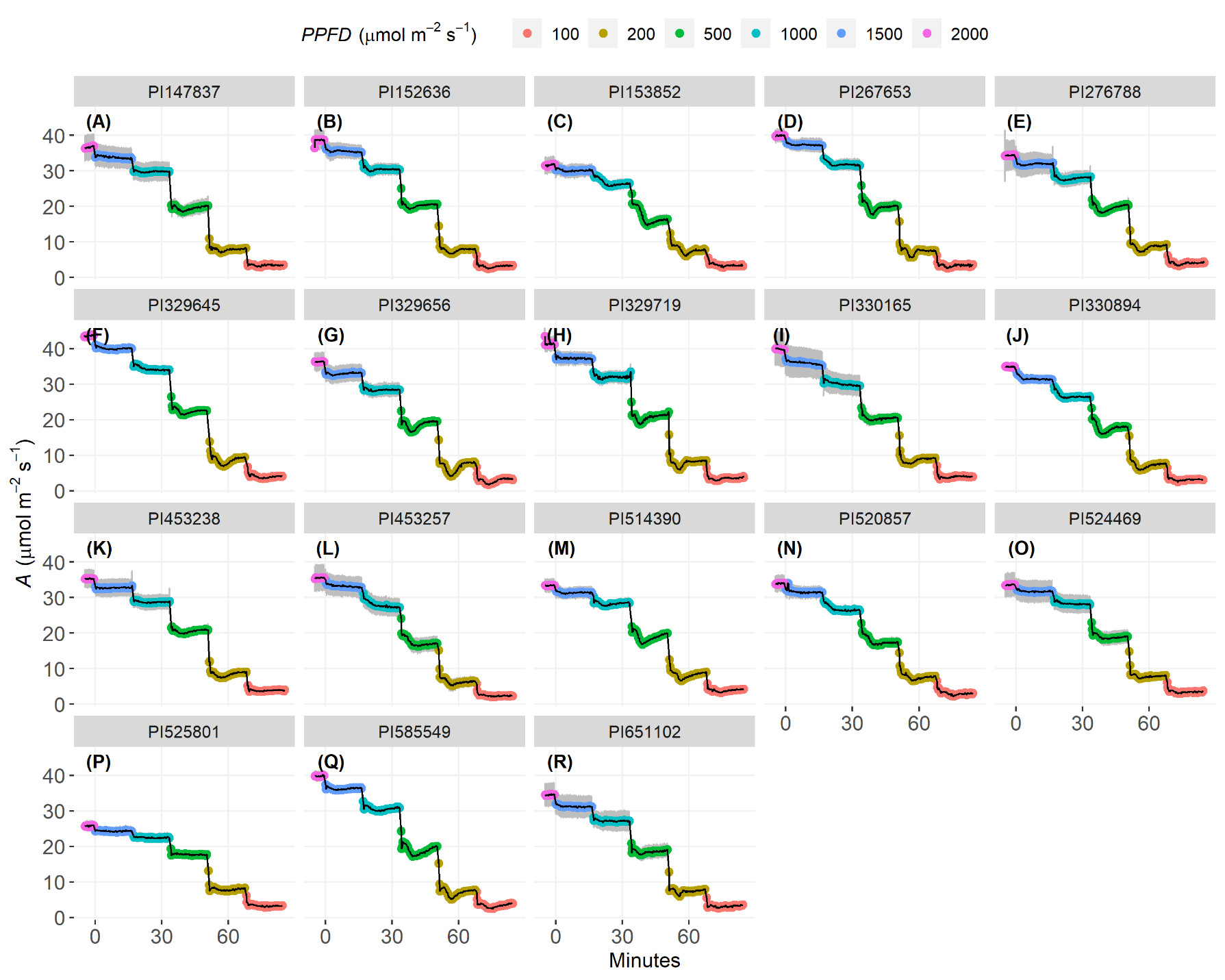


Supplementary Fig. S2: Timecourses of steady-state *A-PPFD* response curves in all accessions. For each curve, leaves were acclimatized to *PPFD* of 2000 µmol m^-2^ s^-1^ for 1 h, then *PPFD* declined in steps every 15 minutes. Each point is a mean ± s.e. of 3-5 plants.


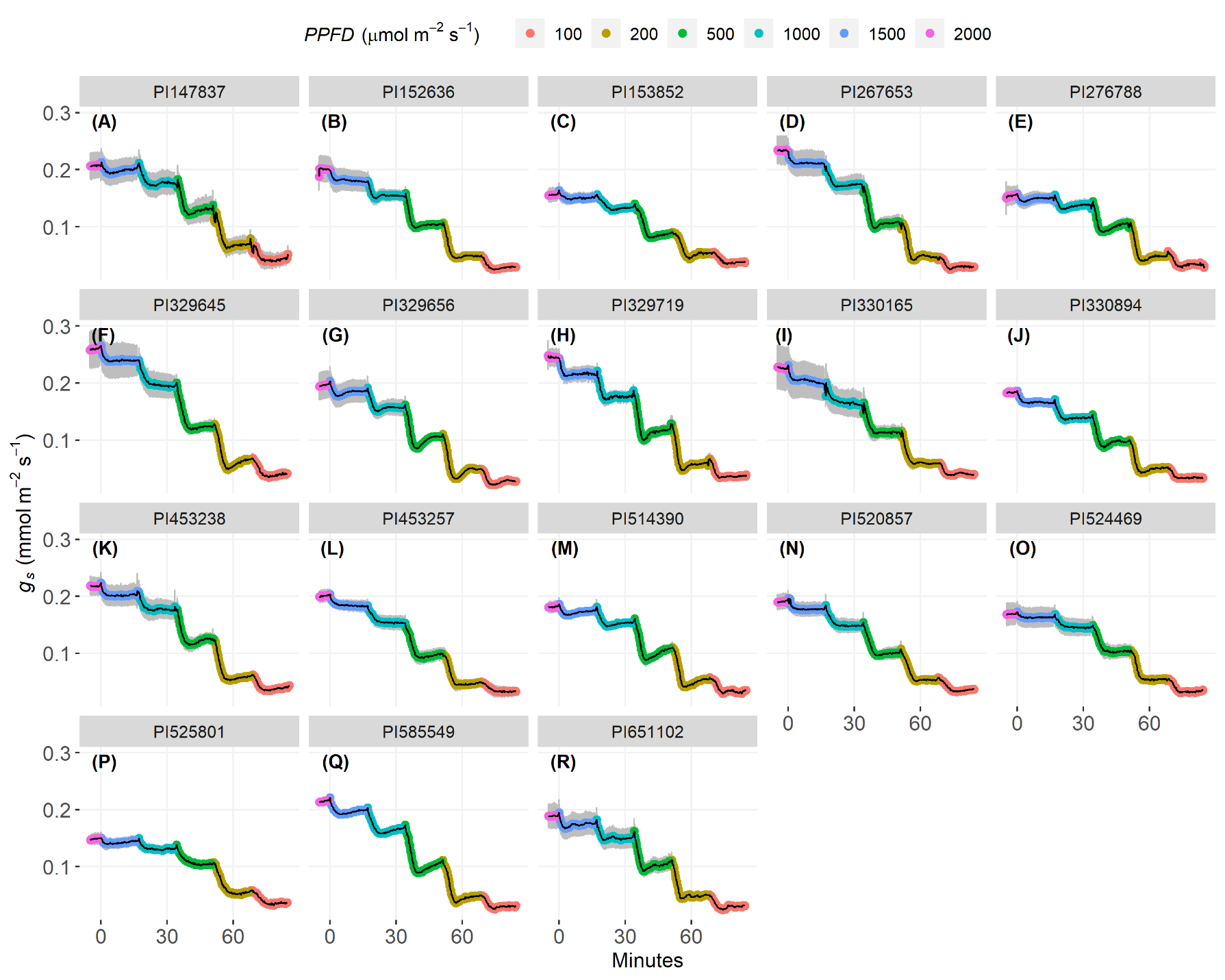


Supplementary Fig. S3: Timecourses of steady-state *g_s_-PPFD* response curves in all accessions. For each curve, leaves were acclimatized to *PPFD* of 2000 µmol m^-2^ s^-1^ for 1 h, then *PPFD* declined in steps every 15 minutes. Each point is a mean ± s.e. of 3-5 plants.


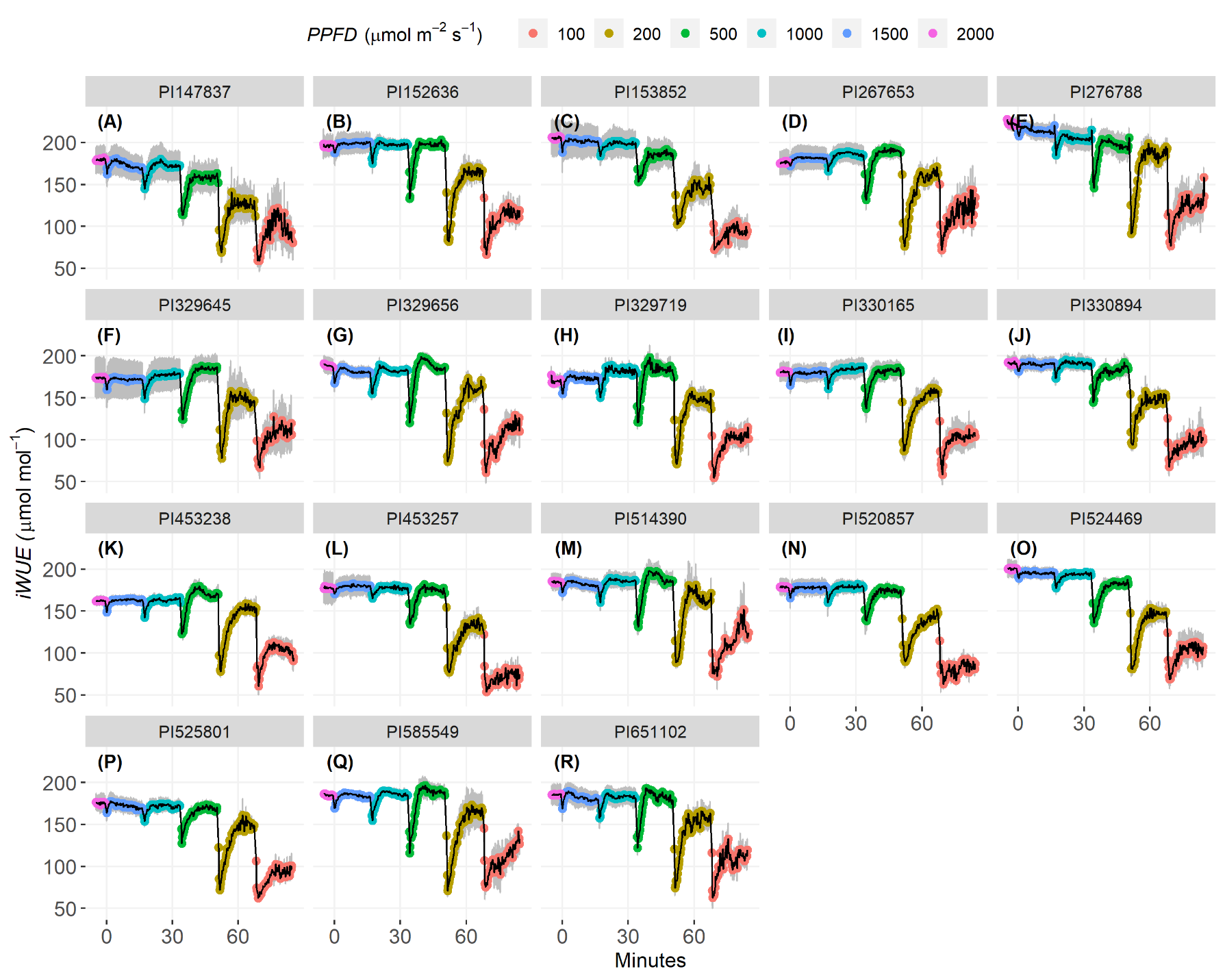


Supplementary Fig. S4: Timecourses of steady-state *iWUE-PPFD* response curves in all accessions. For each curve, leaves were acclimatized to *PPFD* of 2000 µmol m^-2^ s^-1^ for 1 h, then *PPFD* declined in steps every 15 minutes. Each point is a mean ± s.e. of 3-5 plants.


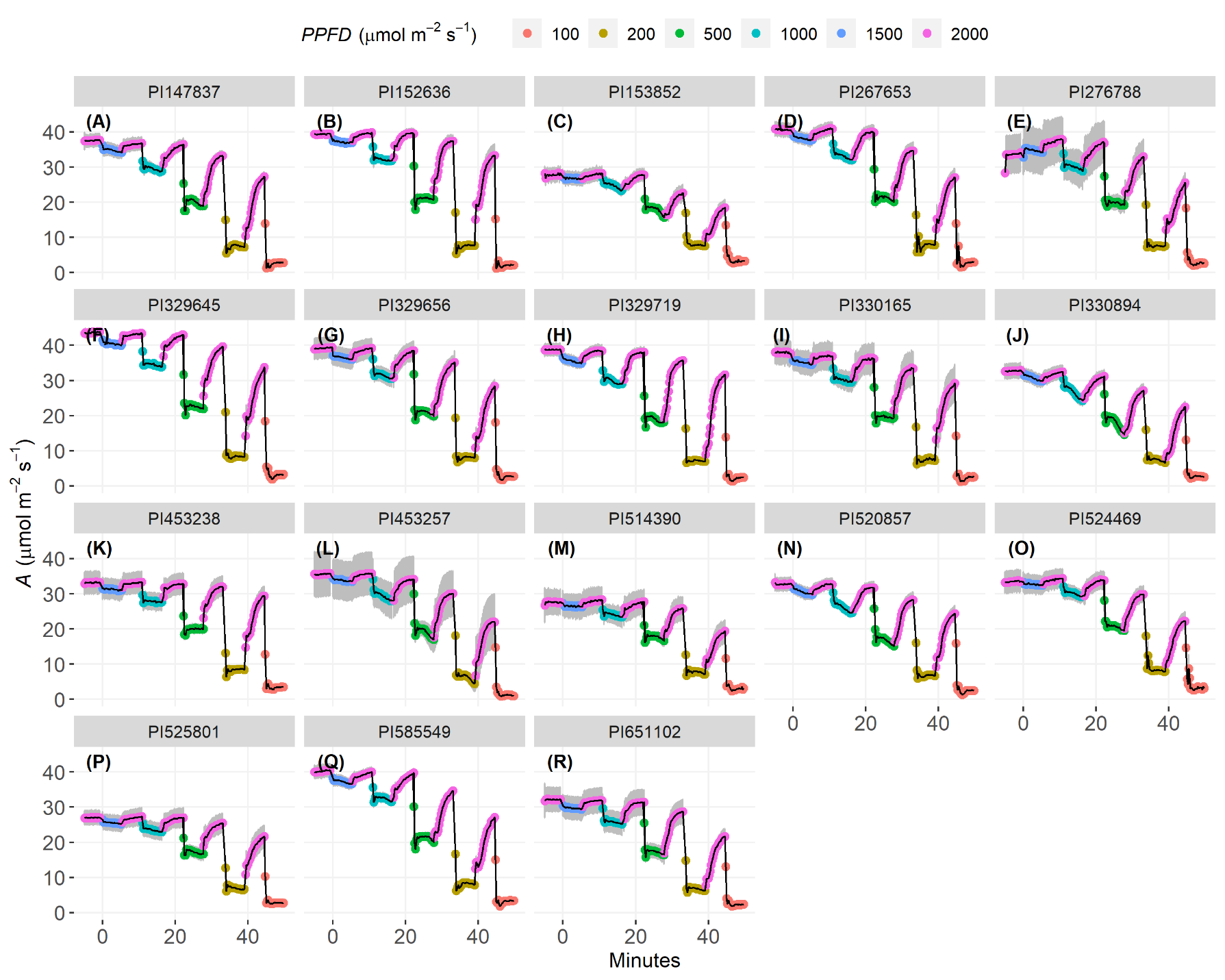


Supplementary Fig. S5: Timecourses of fluctuating *PPFD* response curves for *A* in all accessions. For each curve, leaves were acclimatized to *PPFD* of 2000 µmol m^-2^ s^-1^ for 1 h, then *PPFD* cycled between saturating and non-saturating *PPFD* every 5.5 minutes. Each point is a mean ± s.e. of 2-6 plants.


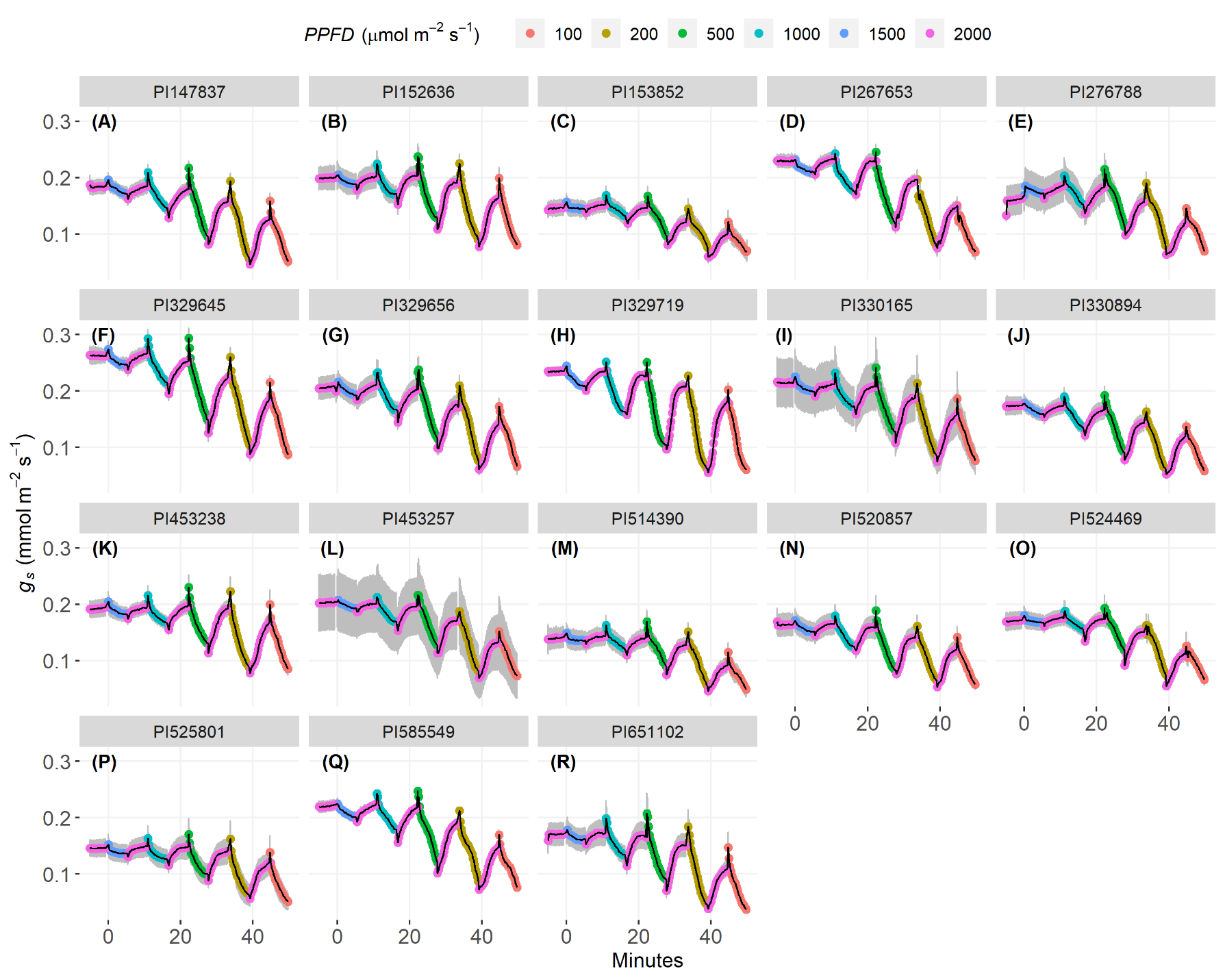


Supplementary Fig. S6: Timecourses of fluctuating *PPFD* response curves for *g_s_* in all accessions. For each curve, leaves were acclimatized to *PPFD* of 2000 µmol m^-2^ s^-1^ for 1 h, then *PPFD* cycled between saturating and non-saturating *PPFD* every 5.5 minutes. Each point is a mean ± s.e. of 2-6 plants.


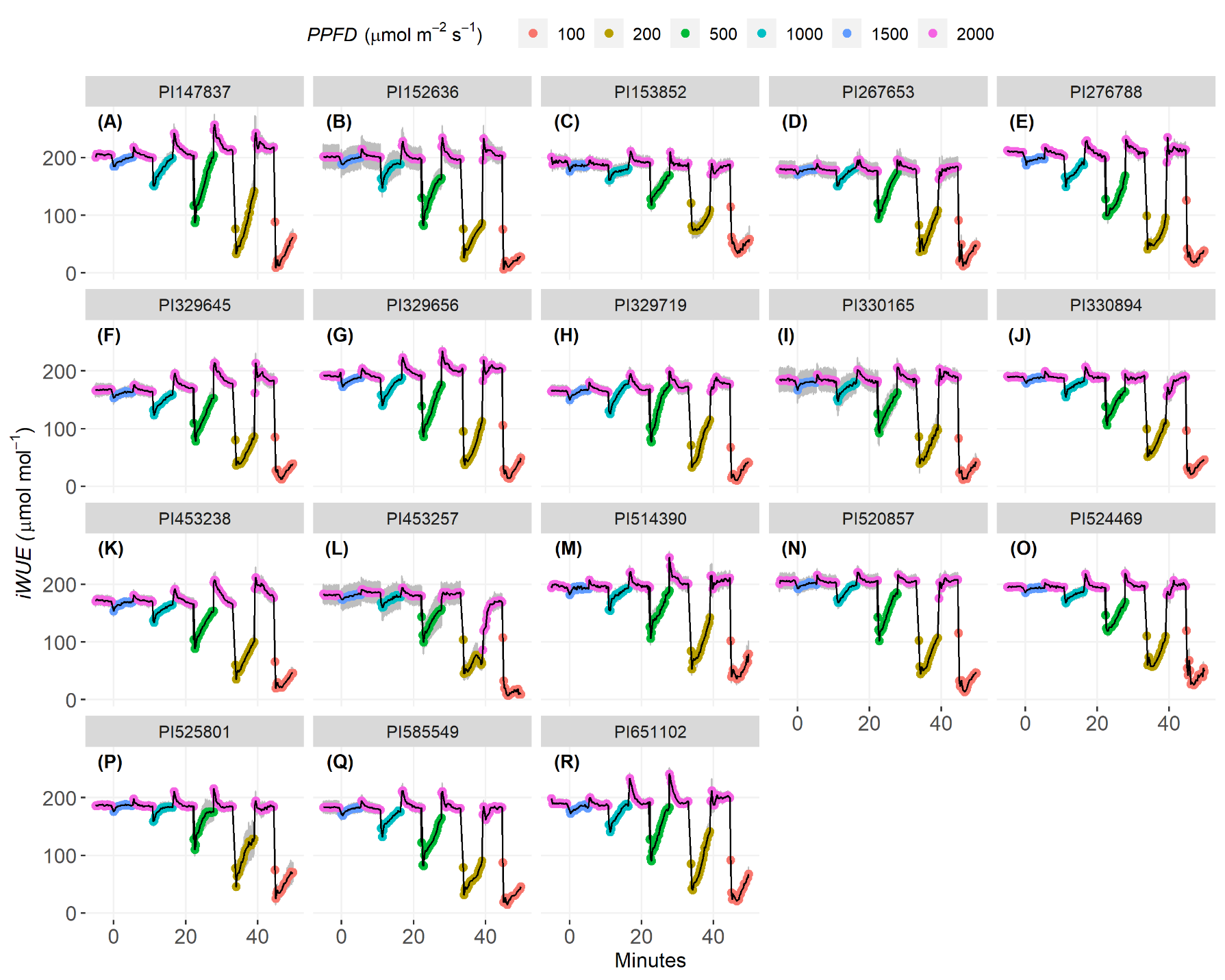


Supplementary Fig. S7: Timecourses of fluctuating *PPFD* response curves for *iWUE* in all accessions. For each curve, leaves were acclimatized to *PPFD* of 2000 µmol m^-2^ s^-1^ for 1 h, then *PPFD* cycled between saturating and non-saturating *PPFD* every 5.5 minutes. Each point is a mean ± s.e. of 2-6 plants.


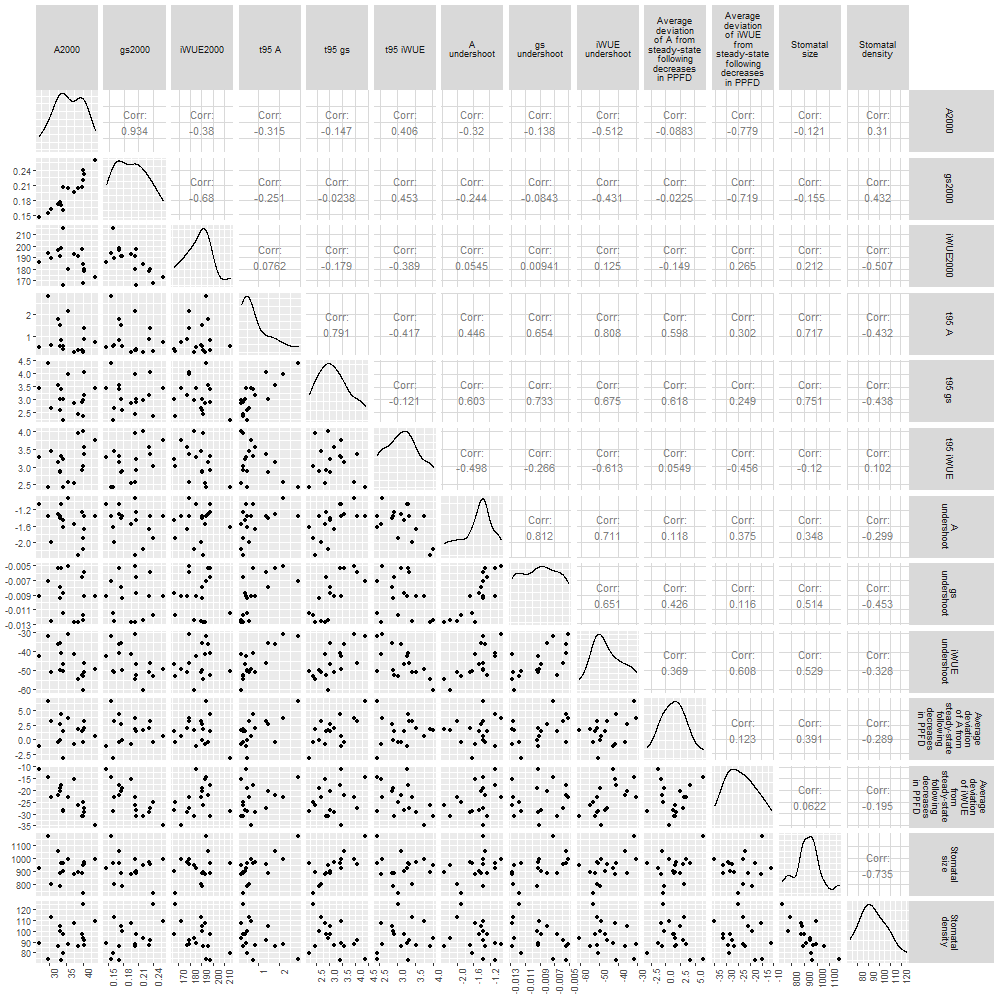


Supplementary Fig. S8: Pearson’s correlation coefficients (*r*, top right panels), pairwise correlation scatterplots (bottom left panels) and density plots (diagonal panels) for traits potentially underlying variation in non-steady-state *iWUE* following decreases in *PPFD*. In scatterplots, each point is a mean per accession. Data are the same as in Figure 6.


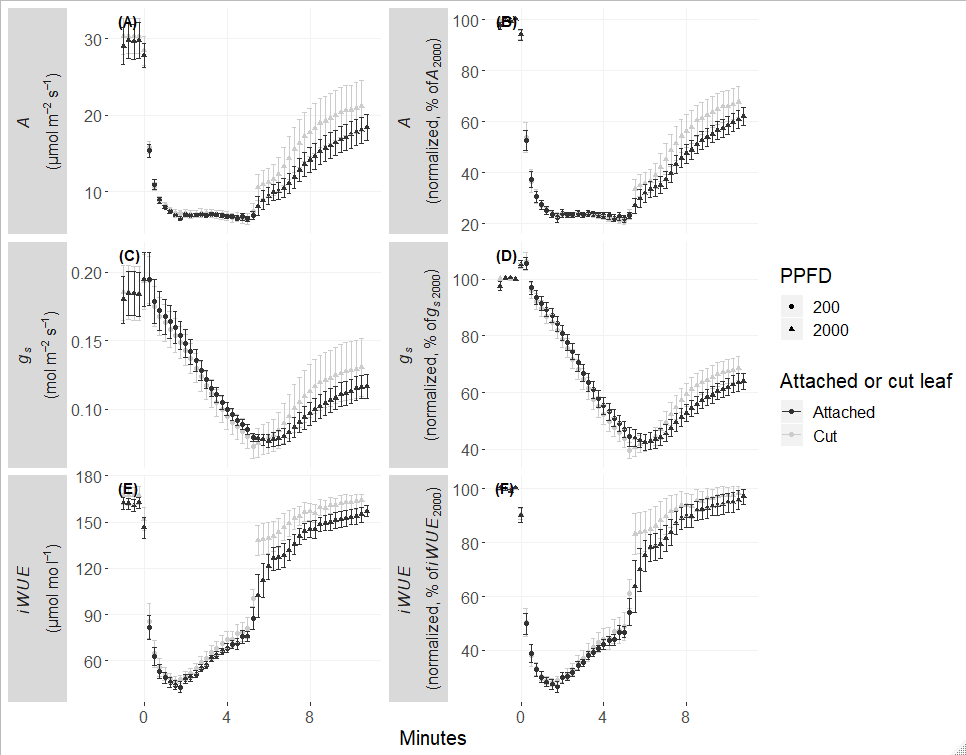


Supplementary Fig. S9: Test of the effect of leaf excision on steady-state and non-steady-state leaf gas-exchange. Timecourses of a) *A*, b) *g_s_*, c) *iWUE*, d) *A* normalized to *A_2000_*, e) *g_s_* normalized to *g_s_* *_2000_*, f) *iWUE* normalized to *iWUE_2000_*. For each curve, leaves were acclimatized to *PPFD* of 2000 µmol m^-2^ s^-1^ until they reached steady-state, then *PPFD* was set to 200 µmol m^-2^ s^-1^ for 5.5 minutes, then back to 2000 µmol m^-2^ s^-1^ for 5.5 minutes. Each point is a mean ± s.e. of 6 plants. Data was collected as follows: on September 1 2019, 12 plants were randomly selected from an irrigated plot of sorghum (cv. TX430). The youngest fully expanded leaf was measured on all plants: 6 were measured while still attached to the plant, and 6 were first removed from the plant as described above. Each leaf was placed in the cuvette of a portable photosynthetic gas-exchange system (LI-6400XT), with *PPFD* set to 2000 μmol m^-2^ s^-1^, block temperature to 25 °C, flow rate to 700 µmol s^-1^, [CO_2_] in the sample cell to 400 ppm and leaf-to-air water vapor pressure deficit maintained at <2 kPa. Once gas-exchange had reached steady-state, *PPFD* was decreased to 200 μmol m^-2^ s^-1^ for 5.5 minutes, then increased to 2000 μmol m^-2^ s^-1^ for 5.5 minutes. Measurements were logged throughout every 5s.
